# Supplementary material for: GroopM: an automated tool for the recovery of population genomes from related metagenomes
Source: PeerJ. 2014 Sep 30;2:e603. doi: 10.7717/peerj.603 (PMC4183954; doi:10.7717/peerj.603)
Supplement: Supplemental Information — The Supplementary materials file contains all supplementary methods, notes, figures, and tables referenced in the main text. [file peerj-02-603-s002.docx]

**Supplementary Materials**

**GroopM: An automated tool for the recovery of population genomes from related metagenomes**

Michael Imelfort, Donovan Parks, Ben J. Woodcroft, Paul Dennis, Philip Hugenholtz and Gene W. Tyson

| **Supplementary File** | **Title** |
| --- | --- |
| Supplementary Methods | Description of the GroopM algorithm |
| Supplementary Note 1 | Justification for using error-free reads in the synthetic data |
| Supplementary Note 2 | GroopM errors |
| Supplementary Note 3 | TF-ESOMs do not accurately define bin boundaries |
| Supplementary Note 4 | Coverage profiles are better indicators of correct bin affiliation  than tetranucleotide frequencies |
| Supplementary Note 5 | GroopM cannot separate genomes from closely related  genotypes into separate bins |
| Supplementary Note 6 | GroopM *Propionibacterium* population genomes  (GM_11 and GM_19) |
| Supplementary Note 7 | Assembly and binning strategies may need to be modified  to suit different experimental designs |
| Supplementary Figure 1 | The synthetic TF-ESOM colored according to the U-Matrix,  bin assignments and corresponding assignment correctness |
| Supplementary Figure 2 | *Propionibacterium acnes* (CARPAC) and *Propionibacterium* sp. (CARPRO) genome bins identified using the two methods |
| Supplementary Figure 3 | Highlighting contigs in GroopM bin GM_34 |
| Supplementary Figure 4 | GroopM plot of GM_34 contigs |
| Supplementary Figure 5 | The effects of using different assemblers to create contigs  for binning with GroopM |
| Supplementary Figure 6 | An illustration of the differences between transformed and  pre-transformed coverage profiles |
| Supplementary Figure 7 | The data transformation process in GroopM illustrated for  a single contig |
| Supplementary Figure 8 | An overview of the process used to order flattened  dimensions during parsing |
| Supplementary Figure 9 | An overview of the preliminary bin building algorithm |
| Supplementary Figure 10 | Contigs in transformed coverage space before and after  two way contraction |
| Supplementary Figure 11 | An example of Hough partitioning |
| Supplementary Figure 12 | An illustration of how Hough transform-derived groups are  filtered and merged |
| Supplementary Figure 13 | An overview of the algorithm used to merge preliminary bins |
| Supplementary Figure 14 | Four views of a GroopM-based self-organizing map |
| Supplementary Figure 15 | GroopM plot of GM_11 contigs |
| Supplementary Table 1 | Genome completeness and contamination estimates for  the TF-ESOM binning of the synthetic dataset |
| Supplementary Table 2 | Genome completeness and contamination estimates for  the GroopM binning of the synthetic dataset |
| Supplementary Table 3 | A comparison of results for the two binning methods  using the synthetic dataset |
| Supplementary Table 4 | Genome completeness and contamination estimates for  the Sharon et al. binning of the human gut microbiome dataset |
| Supplementary Table 5 | Genome completeness and contamination estimates for  the GroopM binning of the human gut microbiome dataset |
| Supplementary Table 6 | GroopM run time and memory usage |

**Supplementary Methods**

**Description of the GroopM algorithm**

**Stage 1 [parse]: Data preparation, parsing and transformation**

*Before using GroopM*

The algorithms used in GroopM require assembled metagenomic data that has been sourced from at least three related samples. Contigs must be in FASTA format and can be compressed using the gzip program. The accuracy of bin assignments is lower with shorter contig lengths, so it is recommended that contigs <500 bp are removed prior to read mapping. Read mapping files (one for each sampling point) must be in BAM format and be sorted and indexed, preferably using samtools ([Li et al. 2009](#_ENREF_8)).

*Coverage and composition profile calculation*

GroopM calculates and stores each contig’s length, tetranucleotide frequency profile and its sample-specific coverage. The coverage value used in GroopM is the truncated mean coverage (TMC), which is calculated using two steps. For each contig, the mean raw (pileup) coverage and corresponding standard deviation is calculated using the raw coverage at all positions along the contig. The TMC is the mean of raw coverage values within one standard deviation of the mean raw coverage. No adjustments to the TMC are made based on either the length or the composition of any contig. GroopM computes each contigs tetranucleotide frequency profile (T-freq) as previously described ([Teeling et al. 2004](#_ENREF_11)). Each T-freq profile is a 136 dimensional vector, so to reduce computational requirements GroopM mostly works with only the principal components of the T-freqs (T-freqPCs). The principal component analysis of the complete collection of T-freqs is performed during the parsing stage and the principal components required to capture at least 80% of the T-freq variance are stored in the database.

*Data transformation*

The final step in the first stage applies a transformation to the TMC profiles that helps to simplify downstream clustering processes. An example of this transformation is illustrated here using a collection of contigs assembled from three replicate anaerobic digesters (**Supplementary Fig. 6)**. This data set contains three samples allowing pre-transformed coverage profiles to be plotted in three-dimensional space, where each axis represents a sampling point and the coordinates of each contig correspond to their TMC at each sampling point. The clusters appear as “spears” pointing away from the origin (**Supplementary Fig. 6a**). Most of the variability within each cluster lies along the axis of the spear, complicating attempts at clustering based on distance methods directly. The points furthest from the origin in each cluster have a relatively higher coverage across all sampling points indicating that this phenomenon is not caused by problems associated with a single sequencing run.

To rectify this problem, a transformation is applied to the coverage profiles that translates the position of the spears and shortens their lengths while conserving spatial relationships between the points belonging to each spear. The transformation maps the spears that are in N ≥ 3 dimensional space into a three-dimensional space such that they stand perpendicular to the transformed XY-plane. Prior to transformation, each contig is viewed as a vector in N dimensional coverage space, where N is the number of sampling points (**Supplementary Fig. 7a**). The N axes are projected onto the transformed space as a set of N vectors in the transformed XY-plane that divide the 360 degrees of the plane into N equal directions, producing a set of “flattened axes” X_F_, Y_F_, Z_F_, … . The projection of any point in the pre-transformed space onto the transformed space is carried out in two steps. First, the point in the pre-transformed space is projected through the origin onto the hyper plane x + y + z + … + n = 1 and the coordinates of the projected point are recorded (**Supplementary Fig. 7b**). This projection has the effect of stacking all the points from one spear onto a dense region on the hyper plane. The coordinates of each projected contig in the pre-transformed space are applied to the flattened axes in the transformed space and the final transformed x and y coordinates of each contig are determined by superimposing the contributions of each of the flattened axes (**Supplementary Figs. 7c** and **7d**). The height of each contig above the transformed plane (z coordinate) is calculated by taking the log of the magnitude of the original vector in the pre-transformed space. Finally, the transformed space is linearly scaled to fit within a cube with sides 1000 units long. The combination of axis translation and log scaling has the effect of turning the spears into rounded contig groups that are more easily clustered using distance metrics (**Supplementary Fig. 6b**).

*Axis ordering in the transformed space*

The clustering methods used by GroopM are based on distance measures in the transformed coverage space where each flattened axis in this space relates to one of the BAM files supplied to GroopM for parsing. GroopM determines the ordering of axes by identifying which BAM files encode the most similar coverage profiles and placing their corresponding axes side by side in the transformed space. This improves reproducibility and prevents ordering artifacts such as placement of two similar samples on axes in opposing directions which will move contigs towards the origin of the transformed coverage space and confound binning efforts. The large number of contigs present in a typical metagenomic assembly makes it necessary to calculate these distances using a subsample of the contigs. GroopM creates this subset in a repeatable and unbiased way by starting with the entire collection of contigs and then repeatedly halving the number of contigs being considered by alternately reordering the contigs by either their average TMC across all samples, or by their first T-freqPC (TFreqPC1) and removing every second contig until the size of the contig set is no larger than 1500 contigs (**Supplementary Fig. 8a**). GroopM then creates a contig-by-sample matrix where each row represents the coverage values associated with a single contig across all samples (**Supplementary Fig. 8b**) and calculates the Manhattan distances for each pair of columns in the matrix. Finally, an “ordering array” is created that initially contains the two samples (columns) with the closest Manhattan distance. The remaining samples are greedily added to the ordering array by repeatedly finding the sample which is closest to the last sample in the array and appending it to the end (**Supplementary Fig. 8c**). The axes are placed in the XY-plane of the transformed coverage space as previously described, anticlockwise according to their order in the ordering array. This fixed ordering is such that axes that point in similar directions have similar coverage and T-freqPC profiles, reducing the number of contigs that lie in the center of the transformed coverage space.

**Stage 2 [core]: Building preliminary bins**

When the transformed coverage space is visualized (with contigs colored based on their GC content), individual population genomes typically appear as like-colored clusters of contigs. The objective of the preliminary bin building stage (coring) is to identify and group together contigs (with similar T-freq profiles) that lie close to the center of each cluster. These preliminary bins will be used as seeds in the contig recruitment stage so the initial clustering is strict, only grouping together contigs that have highly similar coverage profiles, T-freqs and lengths. GroopM builds preliminary bins using only contigs that are at least 1500 bp long, however this value can be modified by the user. The preliminary bin building process iterates through a number of steps (detailed below) until it is no longer possible to make any new preliminary bins (**Supplementary Fig. 9**).

*Determining search area*

Each iteration of the preliminary bin building process begins by selecting the point on the transformed coverage space XY-plane corresponding to the “densest” collection of contigs. This point is found using a square matrix M (side = 1000 units) that stores the *contig density field*. Each contig corresponds to one cell m in M whose position is found by truncating the contig’s x and y coordinates to the nearest integer. The values stored in m and its eight immediate neighbor cells (there are fewer neighbors for edge or corner cells) are increased according to the following formulae:

Amount added to m: log_10_(contig length)

Amount added to four directly adjacent neighbors: 0.6 x log_10_(contig length)

Amount added to four diagonal (corner) neighbors: 0.2 x log_10_(contig length)

Thus each cell in M corresponds to a region in the XY-plane of transformed space and the value in each cell in M is influenced by a combination of the lengths and number of contigs found in the vicinity of its corresponding region in transformed coverage space. At the start of each coring iteration, a Gaussian blur (radius = 2 units) is applied to the values of M producing a “blurred” matrix G, and the x and y coordinates of the densest point in transformed coverage space are found by noting the position of the cell within G with the largest value. GroopM selects all contigs lying above a square region (side = 90 units) centered at this point for further processing. It is important to note that each contig can only be included in one preliminary bin, thus binned contigs are removed from M (and hence also G) before the start of the next coring iteration by applying the reverse of the process described above.

*Contig partitioning within the search area*

The contigs within the search area identified in the previous step typically originate from multiple populations that cannot be reliably separated further using coverage profile information alone. At this point GroopM begins incorporating information about T-freqPC profiles. Contig partitioning within the search area consists of two steps: i) a custom clustering process which draws similar contigs into “tighter”, more distinct clusters (two way clustering), and ii) an iterative algorithm that identifies contig groupings and partitions the tightened clusters (Hough partitioning).

*Two-way clustering*

GroopM uses a custom algorithm called *two-way clustering* to draw contigs with similar transformed coverage and T-freqPC together in order to aid downstream partitioning (**Supplementary Fig. 10**). Groups of contigs with similar profiles in both transformed coverage and T-freqPC space are drawn together by identifying each contigs neighbors in one space (e.g. transformed coverage) and moving that contig towards those neighbors in the other space (e.g. T-freqPC). The amount and direction of movement for each contig C in once space (Space A) is calculated as follows: first, all contigs within an epsilon distance to the contig in the other space (Space B) are labeled as the contig’s neighbors, where epsilon is the median of the Manhattan distances in Space B between each contig and its 10^th^ closest neighbor (for bins with N<10 contigs, the median of the N/2 neighbor is used). The center of the group of neighbors in Space A is found by averaging their coordinates and the direction and magnitude of movement for the contig in this space is towards this central point and 10% of this distance respectively. Contigs are only moved after the magnitude and direction of movements for all contigs in both the transformed coverage and T-freqPC spaces have been calculated. Note that these updated transformed coverage and T-freqPC profiles are temporary and are used only in the partitioning step detailed below. Using an epsilon criterion instead of a k-nearest neighbors approach ensures that contigs in dense or sparse areas will be influenced by large or small numbers of surrounding contigs respectively. Any contigs without neighbors in either space are identified and removed, unless the contig is longer than 1Mbp.

*Hough partitioning*

GroopM uses a novel algorithm called *Hough partitioning* to identify boundaries between contig groups. This algorithm is based on computer vision techniques and is able to identify naturally occurring groupings within the data without requiring any prior estimates of the correct number of groups. Hough partitioning works by representing contigs as pixels in an image in such a way that naturally occurring groups appear as straight horizontal lines. The Hough transform ([Hough 1962](#_ENREF_4)) is used to identify these straight lines, each of which defines a group that includes the contigs whose points formed that line. The image is created by arranging contigs from left to right in ascending order of the measurement being clustered on (i.e. coverage or T-freq values) and vertically based on their dissimilarity to their immediate neighbors, with larger vertical distances between contigs indicating greater dissimilarity. The algorithm can only cluster one-dimensional data, so to cluster based on coverage or T-freq profiles the first principal component of the transformed coverage or T-freqPC1 is used respectively.

Contigs are first sorted in ascending order of the objective measurement. In this example, the objective measurement is the first principal component of transformed coverage (**Supplementary Fig. 11a**). The distance between consecutive contig pairs (Δ) is recorded and each contig is assigned the square of the average of its distance to its two neighbors (Δ_ave_^2^). Averaging affects contigs on the outskirts of a natural grouping, essentially pulling them away from the group’s center, however contigs within a group should be similarly distant to both of their neighbors, thus using Δ_ave_ instead of Δ has little effect. Squaring Δ_ave_ has the effect of shrinking the distance between “close” contigs relative to the distance between “far” contigs. The algorithm works best with square images, so the set of Δ_ave_^2^ values are scaled such that their total sum is equal to the number of contigs being clustered. A list of cumulative sums: [S_1_, S_2_, …, S_n_], where S_n_ is the sum of the scaled Δ_ave_^2^ values for the first n contigs is created and each contig is plotted as a pixel at the unique coordinate (k, S_k_), where k is the position of the contig in the sorted list. This process produces a line graph that rises monotonically and contains one or more discrete “steps”, one for each cluster in the collection of contigs (**Supplementary Fig. 11b**). The equation of the most prominent straight line in the plot is found using the Hough transformation (**Supplementary Fig. 11c**) and the corresponding group of contigs is selected by observing the intersection of this line (with four pixel width) with the pixels on the scatter plot (**Supplementary Fig. 11d**). Following this, the Hough transformation is updated to remove any influence from the newly identified contig group and the process of line identification, contig grouping and transform updating is repeated until all of the contigs have been assigned to some group. Groupings corresponding to horizontal lines represent natural clusters, while groupings corresponding to rising lines that connect two horizontal lines typically contain contigs that lie between two natural clusters and are ignored.

Well assembled populations with small numbers of longer contigs that capture the majority of the genome will typically erroneously cluster with contigs from other genomes or fail to be assigned to a cluster. This problem is resolved by cutting all contigs longer than 5 Kbp into non-overlapping 5 Kbp segments and treating each segment as a single contig so that these long contigs also appear as relatively straight horizontal lines in the image. When several points originating from a single contig fall on two distinct straight lines the contig is assigned to the group corresponding to the line that contains the majority of these points. Contigs originating from a single population that are spread across several consecutive horizontal steps, separated by small jumps, will be incorrectly partitioned into two or more groups. GroopM resolves this potential problem by calculating the gradients of all lines found using the Hough transformation as described above, and discarding any lines with a gradient greater than or equal to one while merging adjacent horizontal lines with a gradient less than one (**Supplementary Fig. 12**).

*Preliminary bin contig recruitment*

This step expands the preliminary bins found in the previous step by recruiting unbinned contigs. The algorithm calculates upper and lower limits for each preliminary bins coverage, T-freqPC and contig length distributions and then recruits all unbinned contigs longer than the preliminary bin contig length cutoff whose profiles fall within their corresponding limits. GroopM calculates the means and standard deviations of the transformed coverage profile, average coverage and T-freqPC distributions for all contigs assigned to each preliminary bin. Five sets of upper and lower cutoffs (one for each dimension of the transformed coverage space, one for average coverage and one for T-freqPC1) are calculated for each preliminary bin. Each cutoff is set at one standard deviation above and below the corresponding mean and all unbinned contigs whose profile values fall within the corresponding cutoff ranges are considered for addition to the bin. Grubb’s test ([Grubbs 1950](#_ENREF_3)) is used to assess whether the length of each potential contig is an outlier to the contig length distribution calculated for the preliminary bin. If the contig length is not an outlier then the contig is added to the bin. Once all bins have been expanded, GroopM removes every preliminary bin with fewer than 10 contigs unless the bin has a cumulative contig length of at least 1Mb. The number of standard deviations used to determine the various profile limits, the minimum number of contigs and minimum cumulative contig length cutoffs can be changed by the user.

*Merging fractured preliminary bins*

The parameters used in the previous steps are deliberately strict and generally produce many instances where population bins are fractured and their contigs are distributed across multiple preliminary bins. The goal of this step is to identify groups of fractured preliminary bins and merge them together. The algorithm consists of three processes: i) identifying pairs of preliminary bins which could be merged (putative merge pairs) based on nearest neighbor calculations, ii) heuristically filtering this list, and iii) merging all preliminary bin pairs that are confirmed as fractured (**Supplementary Fig. 13**).

The object of the first process is to reduce the computational requirements of this exercise. GroopM calculates the median transformed coverage and median T-freqPC for all contigs in each preliminary bin. These median values are used to find the K nearest neighbors to each preliminary bin in both the transformed coverage space and the T-freqPC space. Only preliminary bins that are nearest neighbors in both of these spaces are considered candidates for merging and tested further. A value of K=6 was found to provide a substantial reduction in computation, while maintaining accurate merging of fractured genomes. This process will produce several preliminary bin pairs that must pass a series of tests before being confirmed for merging. First, GroopM calculates the contig length distributions for each preliminary bin in the pair and uses Grubb’s test to evaluate whether the median length of the contigs from the other preliminary bin in the pair is an outlier to this distribution. This approach is based on the idea that contigs belonging to single population genome bins have similar distributions and is more robust and efficient than trying to compare distributions directly. If the median contig lengths for both preliminary bins are not outliers when compared to their respective pair’s distribution then testing continues. The final test in this process involves calculating the three-dimensional bounding ellipsoid of each preliminary bin in the pair in transformed coverage space and independently in the first three PCs of T-freqPC space. Core pairs with overlapping bounding ellipsoids in both these spaces are confirmed and will be merged together. Before ellipsoids are calculated, a heuristic is used to provide an additional reduction in computation time. The heuristic works by immediately merging preliminary bins that, when combined, exhibit a distribution in transformed coverage and T-freqPC spaces typical of an un-fractured preliminary bin. In particular, a preliminary bin pair is confirmed if the median Manhattan distance between contigs from the two preliminary bins in both spaces is less than the median of the median intra-preliminary bin contig distance as measured over all unmerged preliminary bins.

*Automatic preliminary bin refinement using a self-organizing map*

The final step of the preliminary bin creation stage is a refinement process that uses a self-organizing map (SOM) ([Kohonen 1982](#_ENREF_6)). Contigs are represented by four-dimensional vectors, where each vector is a concatenation of the contig’s three GroopM transformed coverage coordinates and its T-freqPC1 value. Each bin is assigned a vector that is the mean of all of its contigs points (bin centroid). The automatic refinement process is carried out in four parts: i) initial SOM training, ii) SOM masking and bin area assignments, iii) second round SOM training and iv) reassignments of contigs using SOMs. First, GroopM creates a standard SOM using bin centroids as the training set (**Supplementary Fig. 14a**). GroopM creates a mask over the SOM surface by summing the difference between each point and its eight immediate neighbors, and masking the points with the greatest total differences. The mask defines a collection of regions on the SOM surface that should have a 1:1 correspondence with the collection of bins, however there are occasions where two or more bins will share a single region (red circles **Supplementary Fig. 14b**). GroopM automatically fixes this problem by lowering the variance cutoff within the region, enlarging the masked area, until a 1:1 correspondence between bins and sub regions is achieved (**Supplementary Fig. 14c**). Finally, the masked SOM is further trained using all the binned contigs as a training set (**Supplementary Fig. 14d**). The extra training round improves classification accuracy for contigs with highly variable coverage profiles or tetranucleotide frequencies. Each contig can only influence the area on the SOM within the region that corresponds to its bin. When this training is complete, all the contigs are stripped of their existing bin assignments and then reassigned using the SOM. Contigs are only assigned to a bin if their lengths, tetranucleotide frequencies and coverage profiles all match the distribution of that bin.

Finally, GroopM determines if any of the refinement steps have created chimeric bins. A bin is marked as ‘likely chimeric’ if the contigs within the bin have an abnormally wide GC distribution. This is determined by modeling the GC distribution of each bin as a normal distribution and the typical variance in GC as a normal distribution calculated over each bin’s variance. Any bin with a Z-score >2 from this GC variance model is marked as likely chimeric.

**Stage 3 [refine]: Refining preliminary bins**

This optional stage allows the user to manually refine the preliminary bins produced in the previous stage by splitting single preliminary bins into multiple pieces, merging preliminary bins together or deleting preliminary bins. GroopM creates an interactive three-dimensional plot of the contigs in transformed space colored based on their GC content, allowing the user to view contig groupings and preliminary bin assignments. Using the UNIX command prompt, the user can specify different plotting options or direct GroopM to split, merge or delete preliminary bins. GroopM splits a preliminary bin into a given number of pieces using the k-means clustering algorithm which clusters contigs based on coverage profiles, T-freq similarity or contig lengths. GroopM displays the contig groupings before and after splitting or merging, and prompts the user for confirmation before completing the operation and saving the updated bin assignments to file.

**Stage 4 [recruit]: Core expansion**

This stage uses the preliminary bins created earlier as templates to recruit any remaining unbinned contigs. First, a SOM is created using the technique described in Stage 2 (core), however in this case the contigs are not stripped of their existing bin assignments. When SOM training is complete, GroopM begins assigning unbinned contigs to existing bins using the method described in Stage 2 above. By default GroopM attempts to recruit all unbinned contigs, however the user can set a lower contig length limit.

**Stage 5 [extract]: Data extraction**

This stage allows the user to extract bins created by GroopM. Each bin can be extracted as a multiple FASTA formatted file containing the collection of its contigs or as a plain text file containing the headers of the reads that mapped to each contig. The user can direct GroopM to extract the entire set of bins or any subset of the whole.

**Supplementary Notes**

**Supplementary Note 1. Justification for using error-free reads in the synthetic data**

This analysis was performed on a single assembly of error-free synthetic reads. The use of error-free reads provides two main advantages: First, the absence of errors in the input read data results in a set of contigs that can be accurately classified via mapping to a set of reference sequences. This is important when determining a verified bin assignment for each contig. Secondly, the use of error-free synthetic reads alleviates to some degree the need to analyze the effects of the software responsible for either read simulation or assembly, allowing for greater focus on the differences and overall performances of the binning methods.

**Supplementary Note 2. GroopM errors**

GroopM is designed to make use of tetranucleotide frequencies when trying to differentiate contigs originating from multiple population genomes with highly similar coverage profiles. If these genomes also have similar tetranucleotide frequencies then GroopM has no means for further differentiation and is forced to group all the contigs into one bin. When only a subset of contigs has this property, GroopM is typically able to separate the majority of contigs into discrete bins however contigs within the tetranucleotide frequency overlap will be assigned to whichever bin has the closest tetranucleotide frequency, resulting in mixing of contigs between bins. The majority of the GroopM errors for the synthetic contigs are of this type. However, from the perspective of this algorithm, these erroneous assignments represent the best possible binning and errors like these are unavoidable in the current implementation.

**Supplementary Note 3. TF-ESOMs do not accurately define bin boundaries**

We recommend caution be exercised when deciding bin affiliations based solely on identifying areas within U-Matrix ridgelines when using TF-ESOMs. To highlight this point we colored the TF-ESOM used in the synthetic data analysis three different ways to highlight the U-Matrix, population bin assignments and the corresponding bin assignment accuracy and show that the majority of errors made using TF-ESOM are clearly clustered together (**Supplementary Fig. 1**). Moreover, these plots highlight multiple cases where simple visual inspection of TF-ESOM topology provides no clear indication of how many population genomes will be contained within any given area delineated by a U-Matrix ridgeline. As illustrated here, a number of seemingly “well behaved” areas contain contigs from multiple organisms. The largest of these in terms of both size and number of assigned bases is bin E_47, dominated by contigs from *Colwellia psychrerythraea*, indicated by the black arrow (**Supplementary Fig. 1b**).

**Supplementary Note 4. Coverage profiles are better indicators of correct bin affiliation than tetranucleotide frequencies**

Boundaries separating population genomes are more clearly defined in plots based on coverage profiles than plots based on tetranucleotide frequencies (**Figs. 2a** and **2b**), indicating that methods that are based primarily on leveraging coverage profile-based information should be able to bin contigs with greater accuracy than those that rely solely on differentiating tetranucleotide frequencies. We quantified how well these different signals indicate correct bin affiliation by calculating the overall average silhouette width (OASW) ([Rousseeuw 1987](#_ENREF_9)) for the true bin assignments of a synthetic metagenomic data set used to validate GroopM. Briefly, the OASW ranges from -1 to 1 and is generally used to indicate cluster correctness with larger values indicating better clusters. For this analysis we interpreted signals producing larger OASW values as being better indicators of bin affiliation. For the synthetic dataset we measured an OASW of 0.302 for coverage profiles and 0.062 for tetranucleotide frequencies, confirming what was apparent via visual inspection. We add that although this analysis is based on synthetic data, the tetranucleotide frequencies are derived from actual bacterial and archaeal reference sequences and the coverage profiles from actual 16S rRNA pyrotag results (see Methods for description of how the synthetic data was produced). Our experience with multiple data sets, including the synthetic data described in this paper, lead us to believe that coverage profiles are generally better indicators of contig origin than tetranucleotide frequencies and that the superior performance of GroopM is due mainly to its ability to leverage these signals.

Contig coverage profiles typically mirror their corresponding organism’s abundance profiles. However, this may not be the case for samples containing closely related species or strain variants whose genomes share large stretches that are identical or nearly-identical. For example, when mapping reads originating from very closely related microbial strains, read mapping programs such as BWA ([Li & Durbin 2009](#_ENREF_7)) will often divide common reads equally between the strains, effectively averaging their coverage profiles and removing the possibility of partitioning using coverage profiles alone. Unfortunately, the high genome similarity which causes coverage profile averaging also produces highly similar T-freqs, limiting the possibility of solving this problem using T-freq profiles. GroopM will not be able to accurately separate these genomes and typically produces one large population genome bin containing contigs from the collection of similar genomes. Inter-genome similarities can also cause assembly software to produce chimeric contigs which will ultimately require reassembly. In these cases, it is arguably more useful to separate these contigs from the remaining metagenome but leave them grouped together for further analysis downstream, which is what GroopM attempts to do.

**Supplementary Note 5. GroopM cannot separate genomes from closely related genotypes into separate bins**

GroopM improves upon composition-only binning approaches by determining bin assignments based primarily on clustering contigs with similar coverage profiles. Sub-groups within each initial coverage profile-based group are found by analyzing tetranucleotide frequencies. Similar regions within closely related genomes can be chimerically assembled, making accurate binning (separation) impossible. Furthermore, correctly assembled contigs from highly similar genotypes often contain near-identical regions, resulting in cross-mapping of reads originating from different organisms that effectively evens out differences in coverage profiles. This in turn causes GroopM to initially group these contigs together, and as similar genotypes will also have similar tetranucleotide frequencies, there is no way that the current implementation of GroopM can separate these contigs into distinct bins. The Sharon method identified four *Staphylococcus epidermidis* strains and corresponding plasmids of which only two strains (1 and 3) had acceptable completeness scores (77% and 83%), while the other two strains contained none of the 111 single copy genes. The strains were produced by using a combination of mapping reads to reference genomes, assembling only reads from single time points and ESOMs. Using GroopM, most of the genomic *S. epidermidis* contigs from the four strains were assigned to bin GM_76 (75.68% complete) while most of the viral contigs were assigned to bin GM_42. The majority of the plasmid contigs were binned into GM_29 and GM_59.

**Supplementary Note 6.  GroopM *Propionibacterium* population genomes (GM_11 and GM_19)**

The closest matches in the Sharon assembly for all of the contigs in bin GM_11 were found in Sharon bin CARPRO. Furthermore, all of these matches had an identity of at least 97%. Contigs from GM_19 most closely matched contigs from Sharon bins CARPAC and CARPRO (**Supplementary Fig. 2a**). The GM_19 contigs that most closely matched contigs in CARPRO had an average identity of only 85% and no match to any of the contigs in CARPAC. Conversely, the GM_19 contigs that most closely matched contigs in CARPAC had an average identity of 99%. Furthermore, these same contigs also matched CARPRO contigs with an average identity of 84% indicating that the contigs that only matched contigs in CARPRO are extra *Propionibacterium acnes* contigs that were not present in the Sharon assembly (**Supplementary Data**). Bin GM_11 has reasonably high contamination (33.33%), however inspection of contigs plotted in transformed coverage space shows no obvious signs of contamination (constrained contig size distribution and low standard deviation of GC measurements) (**Supplementary Fig. 15**). The three contigs at the bottom of this plot contain large regions of self-similarity, possibly due to an assembly error, resulting in a lower than expected coverage score. Moreover, duplication of single copy genes on the two largest of these contigs accounts for 100% of the contamination score for this bin.

**Supplementary Note 7. Assembly and binning strategies may need to be modified to suit different experimental designs.**

Coverage profiles measured over numerous samples offer a powerful alternative to composition-only signals. Interestingly, increasing the number of sampling points can itself cause issues as the technique described here requires co-assemblies that become difficult or impossible to create for large sample sizes. While much progress has been made to reduce the memory footprints and running times associated with complex assemblies ([Bankevich et al. 2012](#_ENREF_2); [Simpson & Durbin 2010](#_ENREF_10); [Titus Brown et al. 2012](#_ENREF_12)) we hold little hope that assembly algorithms will scale to the point of co-assembling the types of large cohorts that are increasingly being produced as part of international metagenomic sequencing projects. The solution to this problem is not immediately clear, although we believe that approaches which combine single sample assemblies or traditional gene-centric analyses ([Karlsson et al. 2013](#_ENREF_5)) with the differential coverage binning methods presented here should be explored. Conversely, the current implementation of GroopM cannot be used for environments where it is not possible to obtain multiple samples. A possible solution to this problem could lie with the development of laboratory-based techniques that operate on partitions of the original sample and can artificially differentially alter the abundances of specific classes of microorganisms in each partition, for example by using different DNA extraction methods ([Albertsen et al. 2013](#_ENREF_1)). Finally, for large data sets it may be necessary to assemble each data set individually and bin each set of contigs using coverage profiles generated from the entire data collection, however this approach requires a further step which integrates contigs from multiple assemblies after binning.


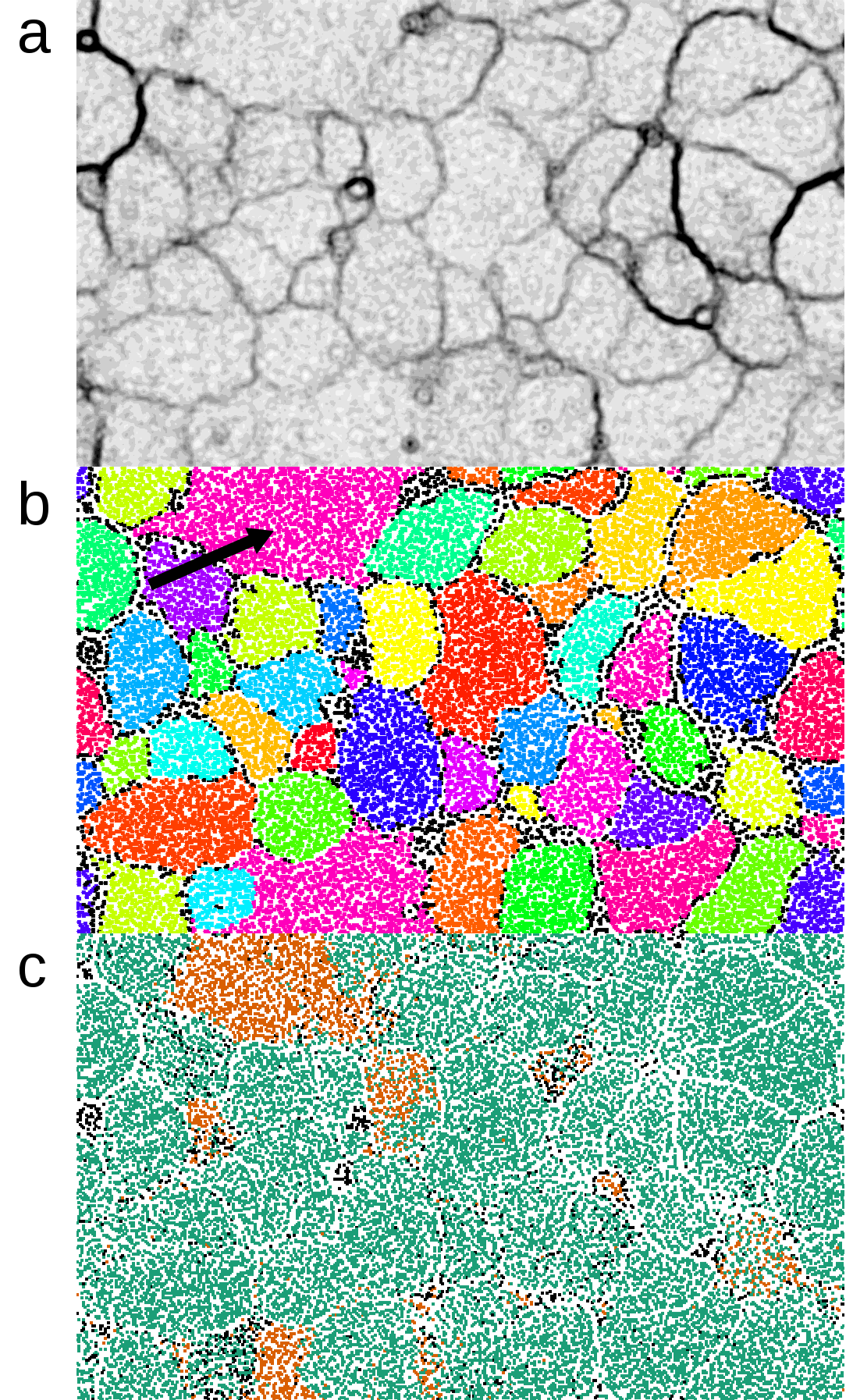


**Supplementary Figure 1.** The synthetic TF-ESOM colored according to the U-Matrix, bin assignments and corresponding assignment correctness. **(a)** The image used when deciding bin boundaries showing ridgelines. **(b)** Contigs (pixels) are colored based on the bin assignments found using the TF-ESOM method. Contigs that could not be assigned to a bin were colored black. **(c)** Contigs are colored based on whether they were assigned correctly (green), incorrectly (orange) or not assigned to any bin (black). This plot clearly demonstrates how most of the incorrectly assigned contigs are localized on the TF-ESOM.


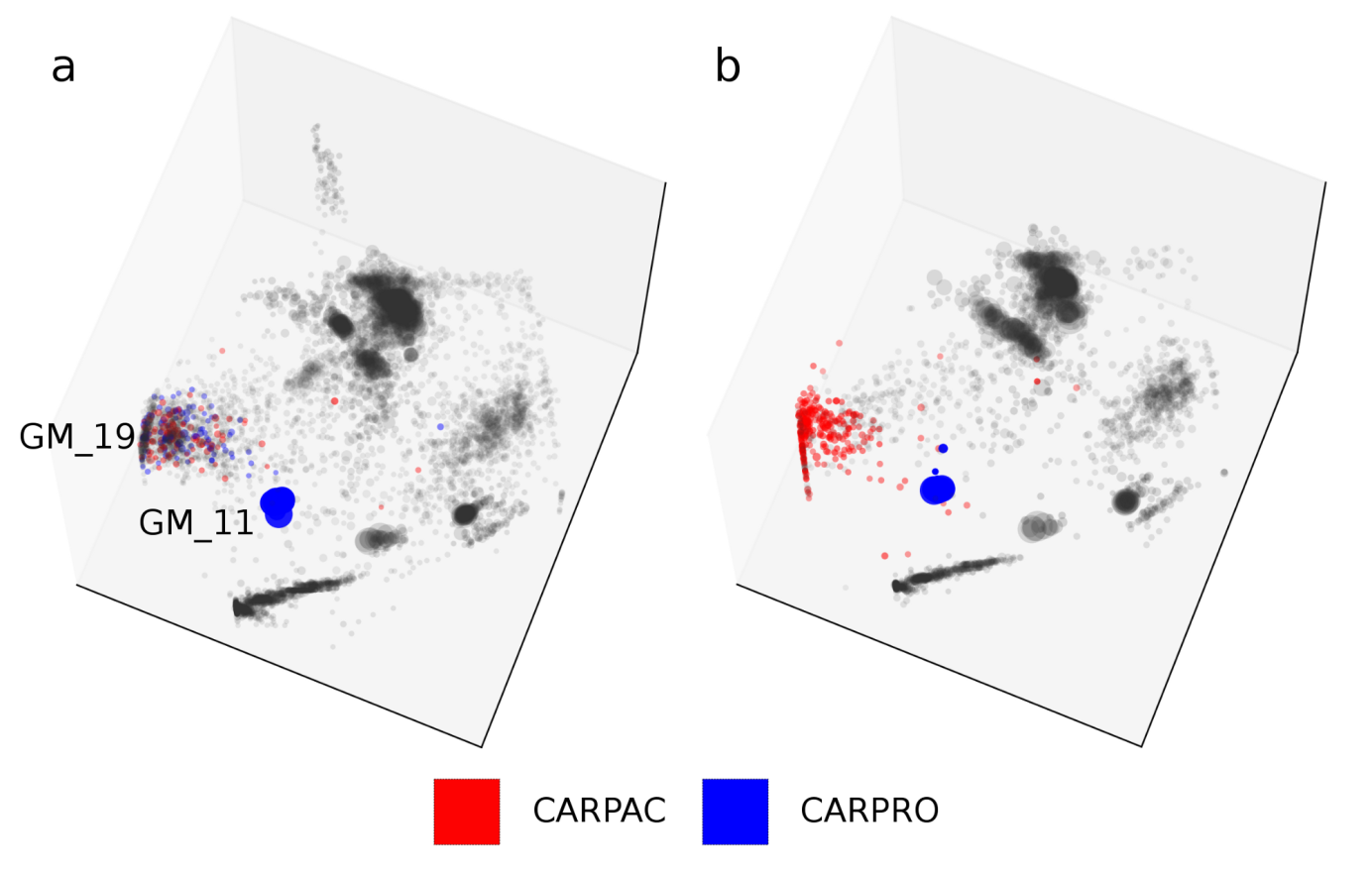


**Supplementary Figure 2.** *Propionibacterium acnes* (CARPAC) and *Propionibacterium sp*. (CARPRO) genome bins identified using the two methods, visualized in GroopM transformed coverage space. **(a)** SPAdes-created contigs are colored according to the bin assignments of their closest matching contigs in the Sharon assembly. Contigs in GM_19 that were not present in the Sharon CARPAC bin are colored blue. **(b)** The Sharon contigs are colored based on their bin assignments.


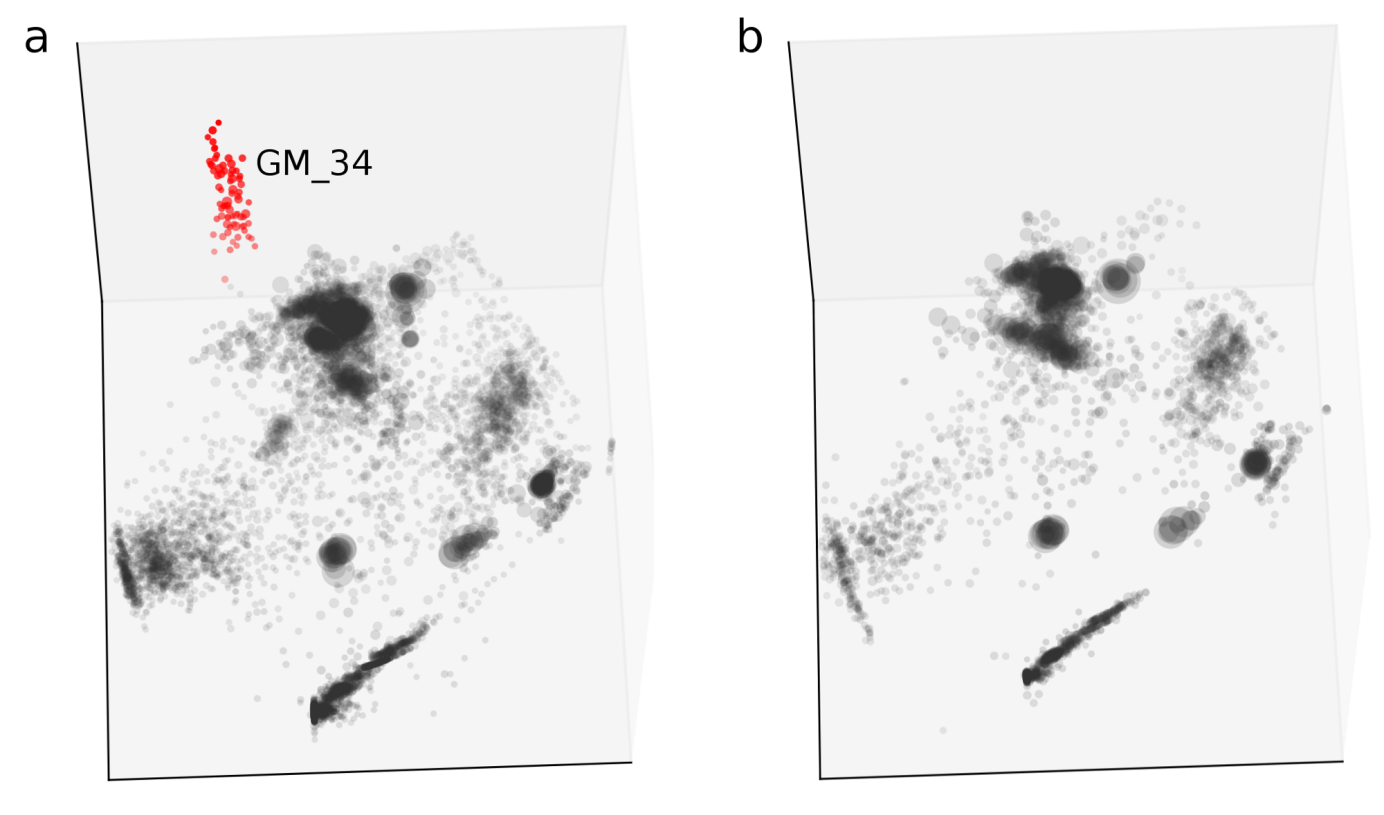


**Supplementary Figure 3.** Contigs from the GroopM and Sharon analyses plotted in GroopM transformed coverage space. **(a)** SPAdes-created contigs belonging to GM_34 (partial *Proteobacteria* genome) are highlighted in red. **(b)** The Sharon assembly contains no contigs in this region.


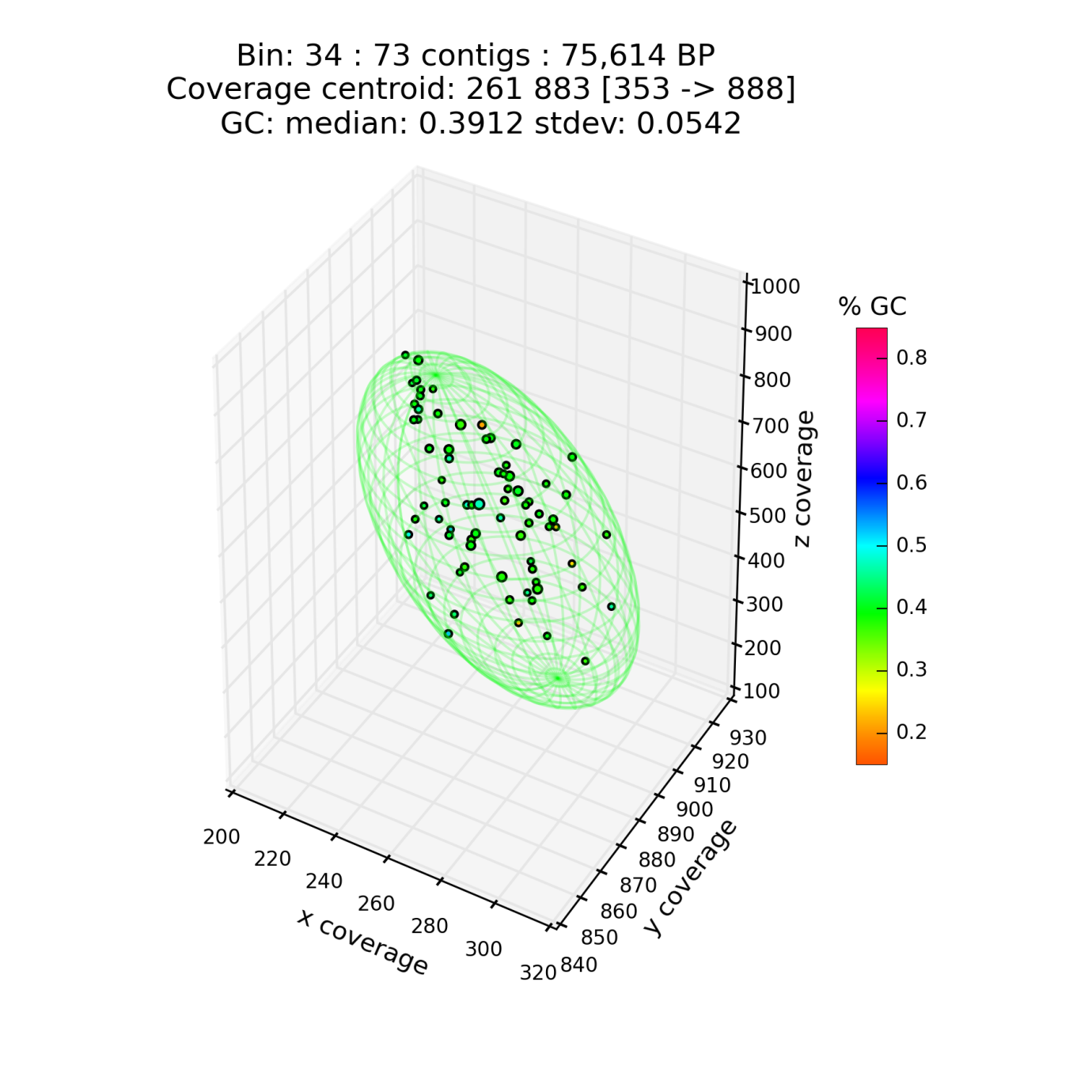


**Supplementary Figure 4.** GroopM plot of GM_34 contigs in GroopM transformed coverage space. The size of each point is proportional to the length of the contig it represents and is colored based on GC content. The relatively stable size distribution, tight clustering and low GC standard deviation indicate that this bin contains minimal contamination.


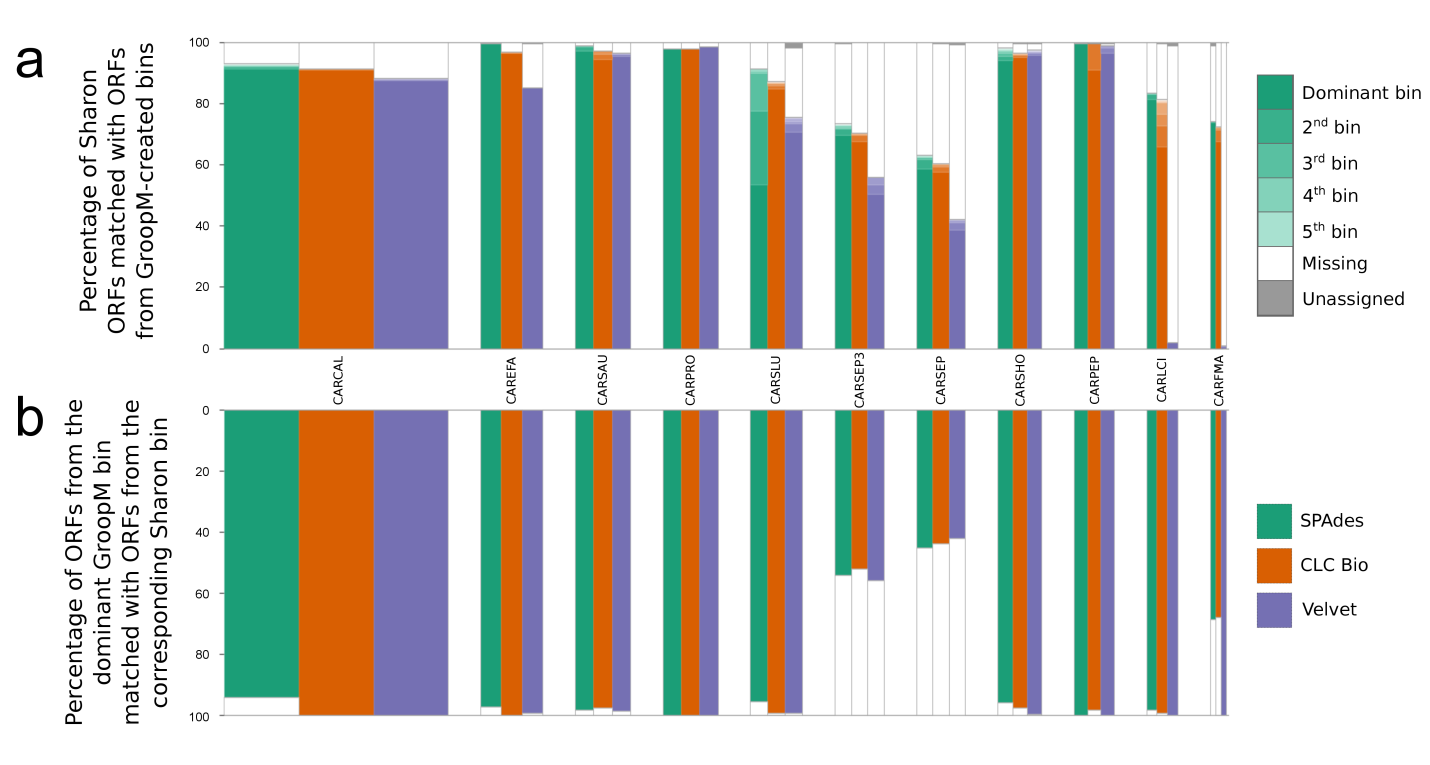


**Supplementary Figure 5.** GroopM bin assignments for the Sharon et al. data generated using three different assemblers. Both graphs show the percentages of open reading frames (ORFs) common between Sharon et al. bins (with at least 1000 ORFs) and their corresponding GroopM-derived bins. Bar widths are proportional to the total number of ORF bases in each Sharon bin, and each assembler is represented using a different color. Within each set of three bars, similar heights (of a single shade) indicate that GroopM has produced similar bins using contigs from the different assemblers. **(a)** The percentage of ORFs (total bases) for each Sharon bin that matched ORFs in GroopM bins. For each Sharon bin, the majority of ORFs matched ORFs from a single GroopM bin (dominant bin, darkest shade) with the remaining ORFs mapping to several other bins (progressively lighter shades). The percentage of ORFs that did not have suitable matches to any ORFs in assembled contig is colored white. ORFs matching contigs unassigned to any bin are colored grey. The majority of these missing ORFs are caused by contig ends truncating ORF boundaries (as opposed to missing contigs) except for CARSEP and CARSEP3 where the missing ORFs are the result of GroopM binning the two strains together (**Supplementary Note 2**). **(b)** The proportion of ORFs (total bases) in the dominant GroopM bin that aligned to its corresponding Sharon bin. A smaller amount of white indicates a lower proportion of contamination in the GroopM bin. The large amount of white for CARSEP and CARSEP3 is again caused by GroopM binning the two strains together.


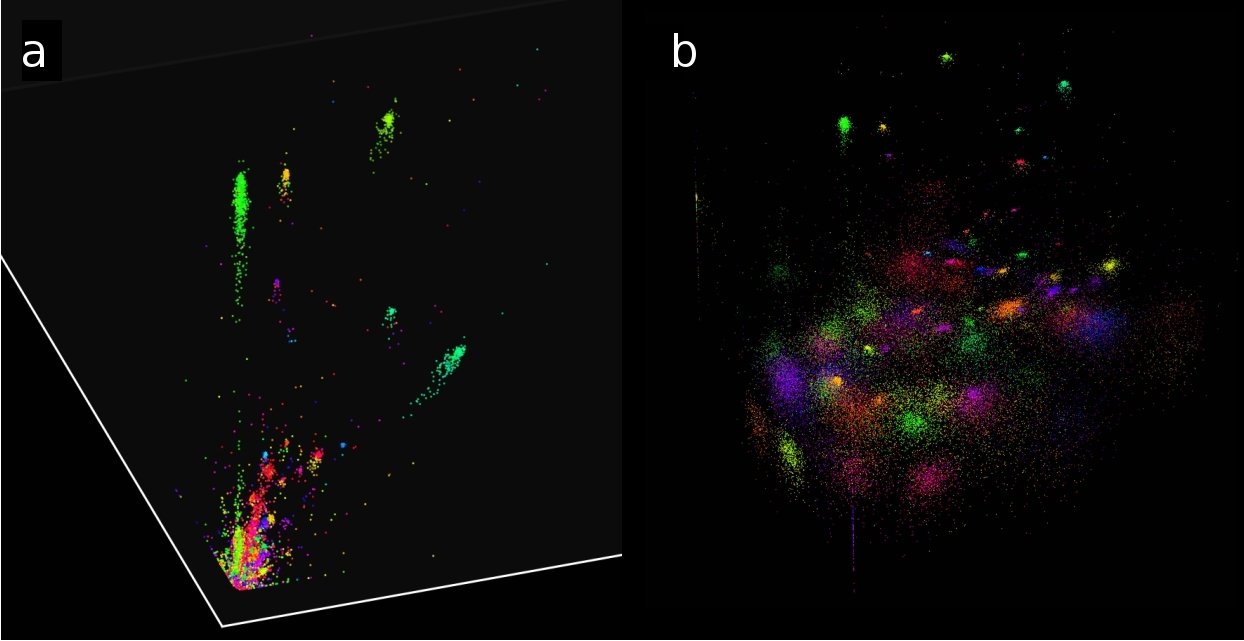


**Supplementary Figure 6.** An illustration of the differences between pre-transformed and transformed coverage profiles. **(a)** Three-dimensional plot of contigs from three spatiotemporally distinct anaerobic digester samples with coordinates based on raw coverage profiles. Contigs are colored based on GC. Each colored cluster of points represents a distinct microbial population genome. **(b)** The same contigs (and coloring) after the GroopM transformation has been applied.


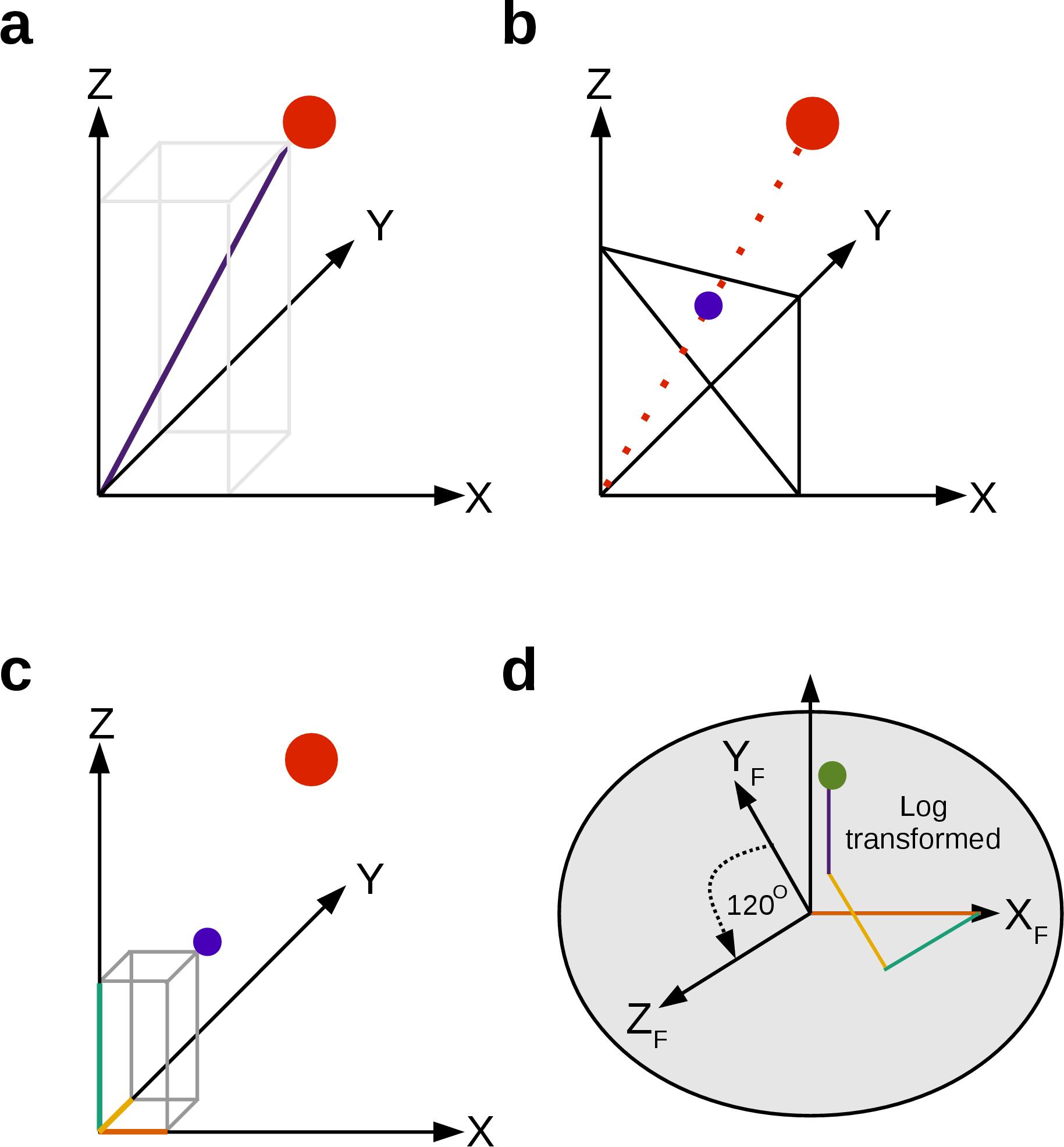


**Supplementary Figure 7.** The data transformation process in GroopM illustrated for a single contig. **(a)** The contig is mapped in N-dimensional coverage profile space before transformation. The magnitude of the position vector for this contigs is recorded. **(b)** The contig is projected through the origin onto the hyper plane X + Y + … + N = 1. **(c)** The coordinates of the projected point are recorded. **(d)** The projected coordinates are applied to the flattened axes (X_F_, Y_F_, Z_F_, …) in the transformed space and superimposed to produce the transformed xy coordinates. The z coordinate in the transformed space is the log transformed magnitude recorded in Panel a.


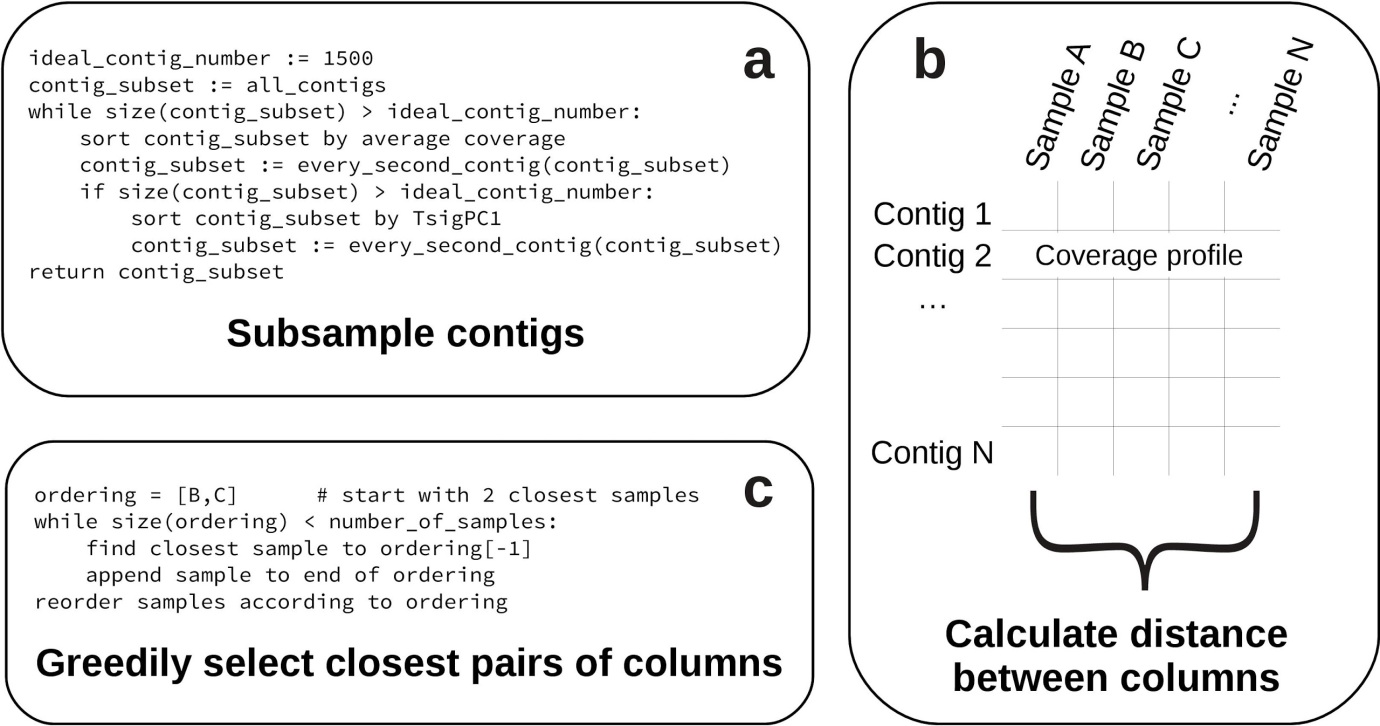


**Supplementary Figure 8.** An overview of the process used to order flattened dimensions during parsing. **(a)** Pseudo code for the procedure used to subsample contigs. **(b)** An illustration of the method used to identify the BAM files that encode the most similar coverage information. **(c)** Pseudo code for the procedure used to reorder the axes based on distances calculated in b.


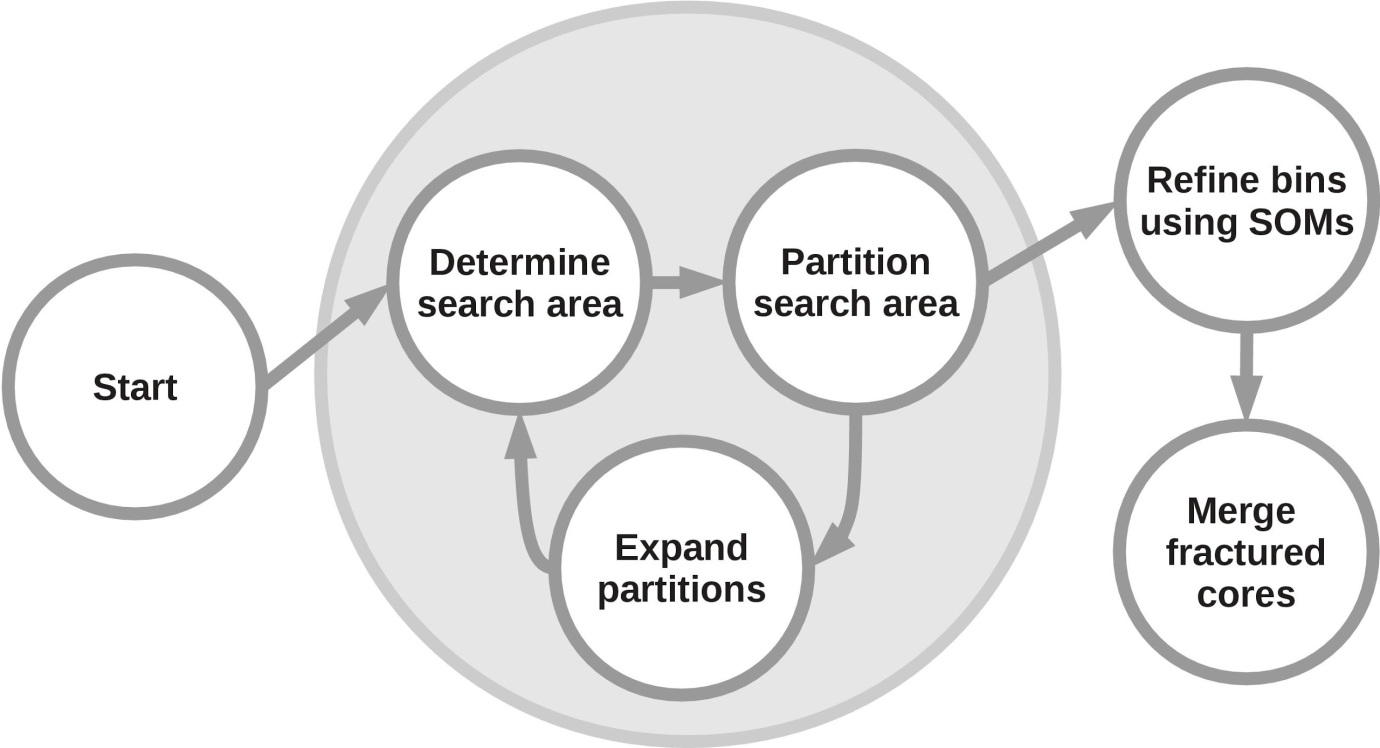


**Supplementary Figure 9.** An overview of the steps carried out within GroopM’s “core” stage (**Fig. 1**). The tasks listed within the grey shaded area are used to produce the initial collection of core bins and are repeated until no more initial bins can be created. These bins are then automatically refined before being merged (where possible) and returned to the user.


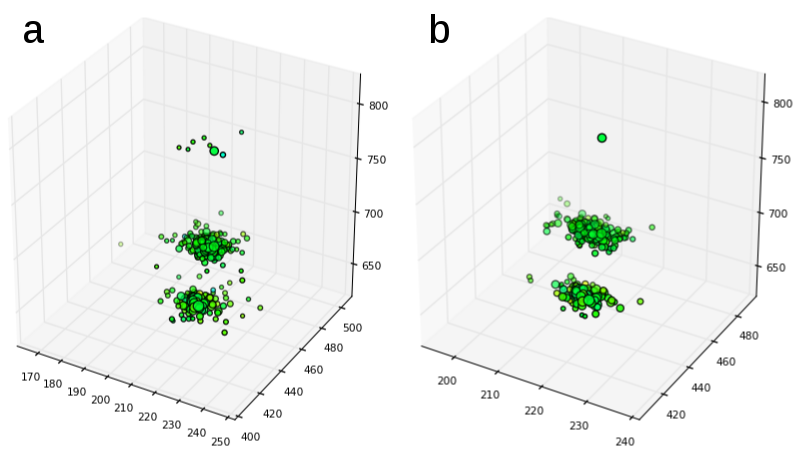


**Supplementary Figure 10.** Contigs in transformed coverage space before **(a)** and after **(b)** two way contraction.


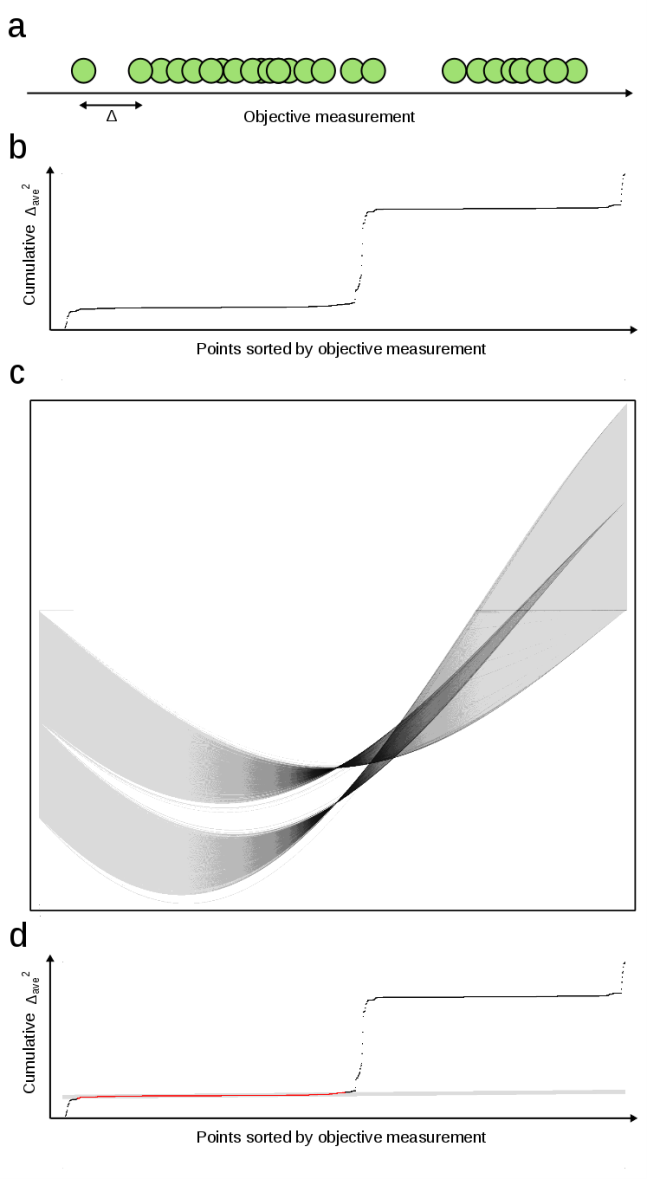


**Supplementary Figure 11.** An example of Hough partitioning using the contigs shown in in Supplementary Fig. 10. **(a)** Points are sorted in ascending order of the objective measurement (in this case coverage), the distance between consecutive pairs of points (Δ) is recorded and each point is assigned the square of the average of its distance to its two neighbors. (Δ_ave_^2^). **(b)** A scatter plot of the index of each ordered point versus the cumulative sum of Δ_ave_^2^ values. The plot is scaled so that it is square. Clusters in the original data appear as straight horizontal lines. **(c)** The Hough transform of the image in Panel b. Each of the two straight lines in Panel b corresponds to one “ribbon” in this image. The Hough transform is used to select the most prominent line in panel b. **(d)** Points colored red lie on the line found using the Hough transform and are interpreted as one cluster. These points are removed from the data before the next Hough partitioning iteration.


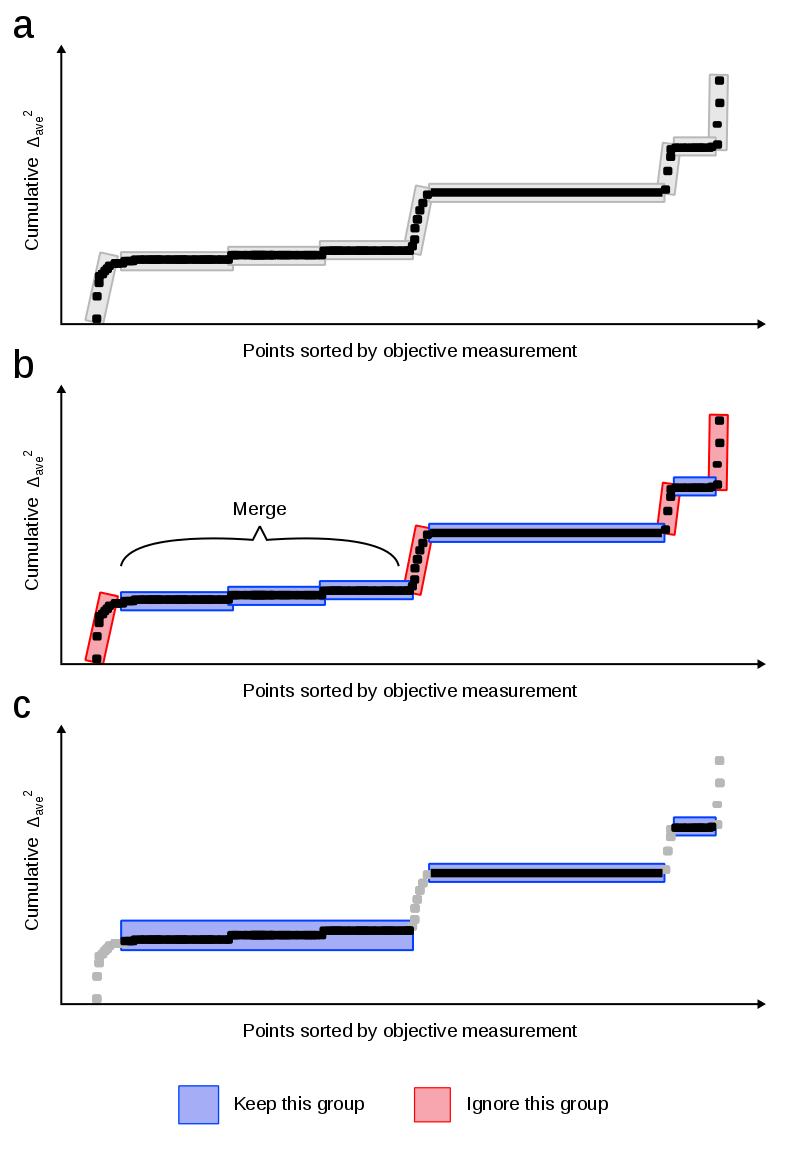


**Supplementary Figure 12.** An illustration of how Hough transform-derived groups are filtered and merged. **(a)** Contigs are plotted in ascending order of the objective measurement as shown in Supplementary Fig. 11b. Grey boxes indicate clusters returned using the Hough transform. The gradient of the line passing through the clusters is calculated and those with a gradient larger than 1 are ignored. All remaining directly adjacent clusters are merged. **(c)** The final clusters after filtering and merging. Contigs that have not been included in any clusters are colored grey.


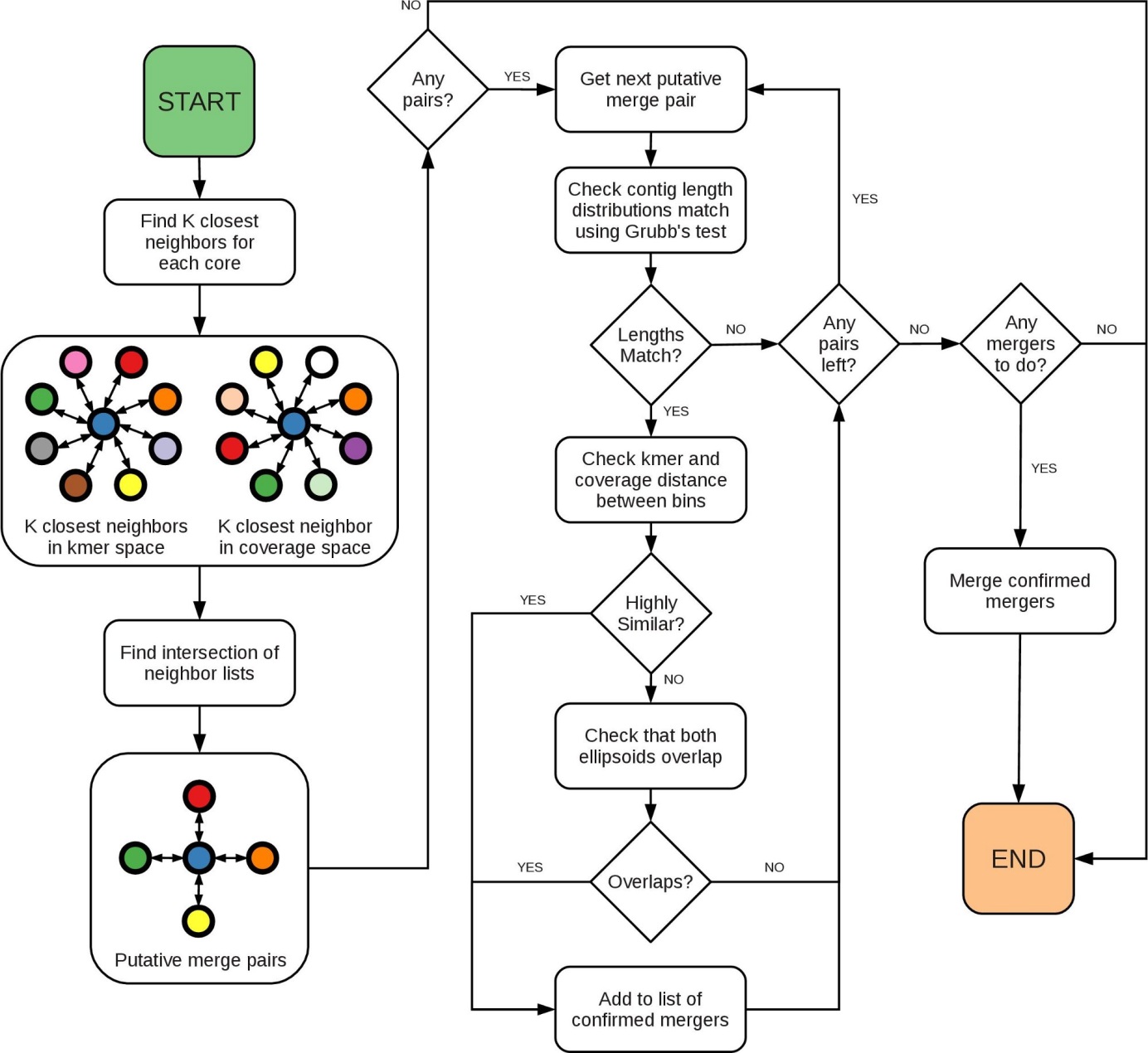


**Supplementary Figure 13.** An overview of the algorithm used to merge preliminary bins.


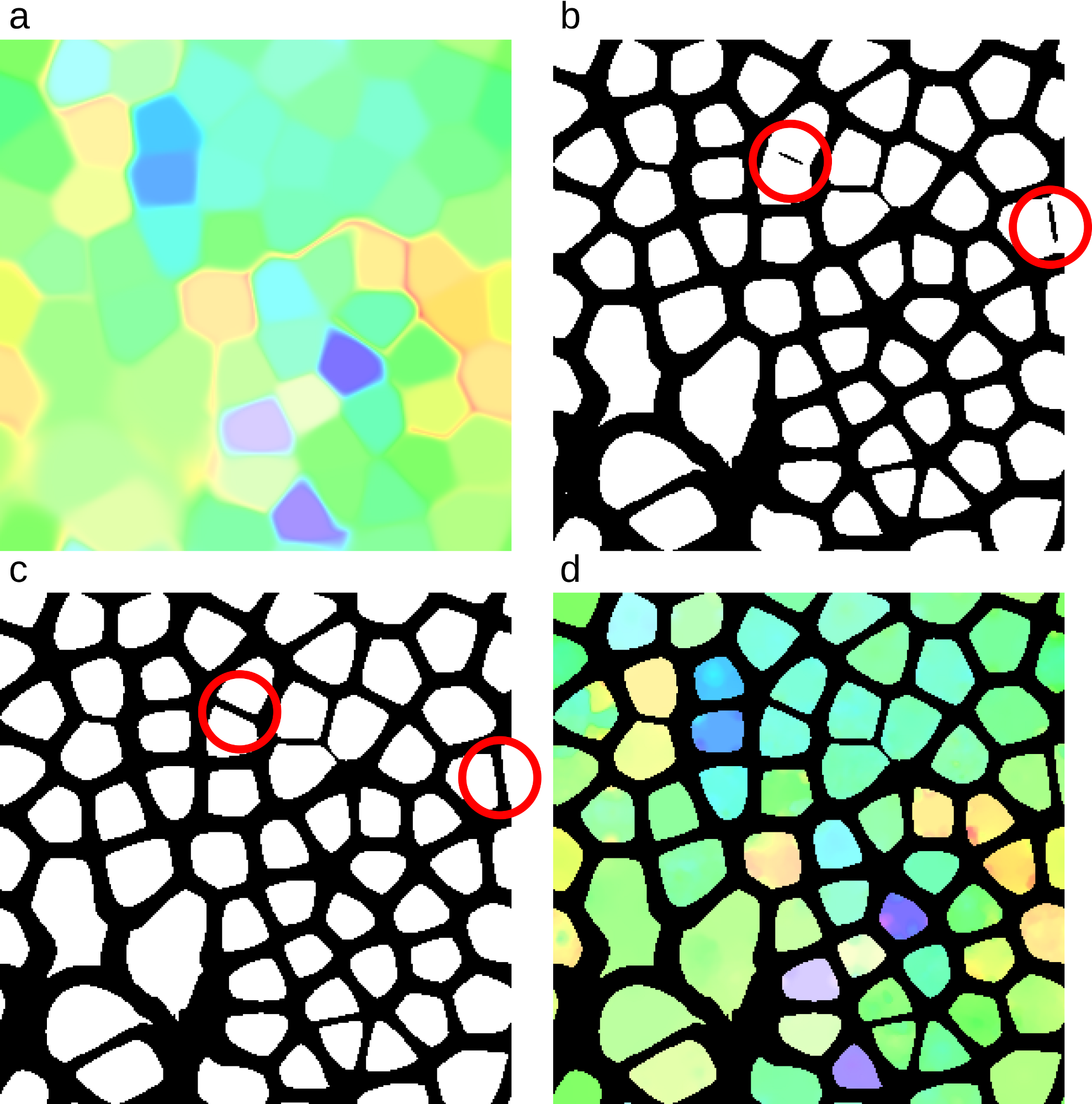


**Supplementary Figure 14.** Four views of a GroopM-based self-organizing map (SOM) and corresponding mask at various steps during training and refinement. **(a)** The SOM after initial training. Each colored region corresponds to one preliminary bin. **(b)** The initial mask which is used to remove ambiguous areas within the SOM. Note the poorly defined boundaries circled in red. **(c)** The same mask after poorly defined boundaries have been repaired. **(d)** The final SOM after secondary training with mask applied.


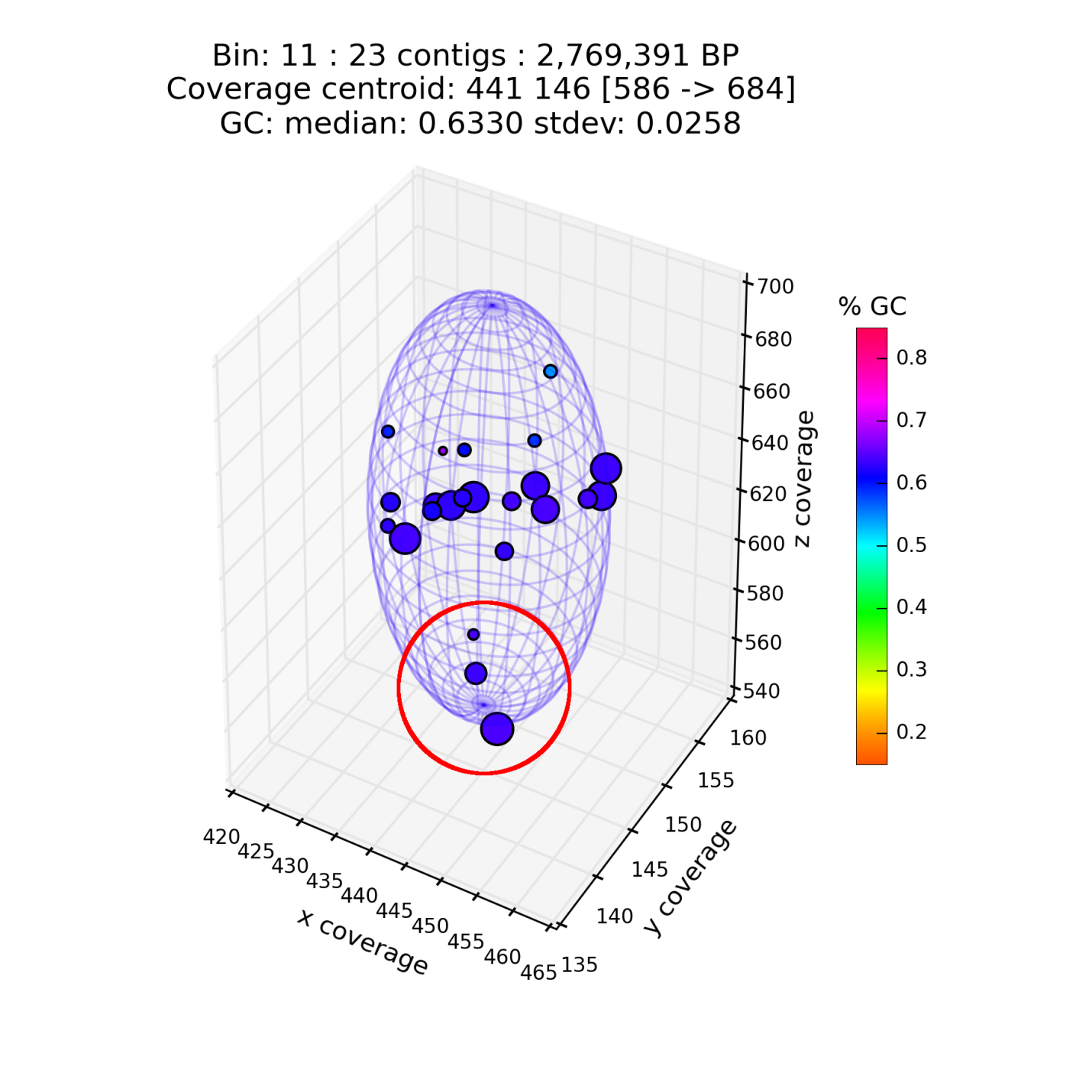


**Supplementary Figure 15.** GroopM plot of GM_11 contigs in GroopM transformed coverage space. Each point’s size is proportional to the length of the contig it represents and its color is based on GC content. The three circled contigs contain large regions of self-similarity, possibly due to an assembly error, resulting in a lower than expected coverage score (which draws them towards the bottom of the image). Moreover, duplication of single copy genes on the two largest of these contigs accounts for 100% of the contamination score for this bin.

**Supplementary Table 1**. Genome completeness and contamination estimates for the TF-ESOM binning of the synthetic dataset. The completeness estimate is based on the proportion of 111 universal single copy maker genes found in the bin. The contamination estimate indicates the multiplicity of these genes.

| **Unique ID** | **Summary statistics** | | **Single copy gene counts** | | | | | |
| --- | --- | --- | --- | --- | --- | --- | --- | --- |
|  | **Completeness** | **Contamination** | **0** | **1** | **2** | **3** | **4** | **5+** |
| E_45 | 95.5 | 93.69 | 5 | 2 | 19 | 67 | 17 | 1 |
| E_39 | 94.59 | 1.8 | 6 | 103 | 2 | 0 | 0 | 0 |
| E_38 | 93.69 | 2.7 | 7 | 101 | 3 | 0 | 0 | 0 |
| E_32 | 93.69 | 0.9 | 7 | 103 | 1 | 0 | 0 | 0 |
| E_37 | 92.79 | 1.8 | 8 | 101 | 2 | 0 | 0 | 0 |
| E_46 | 92.79 | 0.9 | 8 | 102 | 1 | 0 | 0 | 0 |
| E_24 | 92.79 | 6.31 | 8 | 96 | 5 | 2 | 0 | 0 |
| E_1 | 91.89 | 82.88 | 9 | 10 | 91 | 0 | 1 | 0 |
| E_22 | 91.89 | 1.8 | 9 | 100 | 2 | 0 | 0 | 0 |
| E_12 | 91.89 | 0.9 | 9 | 101 | 1 | 0 | 0 | 0 |
| E_30 | 90.09 | 4.5 | 11 | 95 | 5 | 0 | 0 | 0 |
| E_8 | 89.19 | 1.8 | 12 | 97 | 2 | 0 | 0 | 0 |
| E_17 | 86.49 | 0.9 | 15 | 95 | 1 | 0 | 0 | 0 |
| E_36 | 86.49 | 3.6 | 15 | 92 | 4 | 0 | 0 | 0 |
| E_11 | 85.59 | 1.8 | 16 | 93 | 2 | 0 | 0 | 0 |
| E_29 | 83.78 | 3.6 | 18 | 89 | 4 | 0 | 0 | 0 |
| E_28 | 80.18 | 0.9 | 22 | 88 | 1 | 0 | 0 | 0 |
| E_33 | 79.28 | 0.9 | 23 | 87 | 1 | 0 | 0 | 0 |
| E_10 | 76.58 | 0.9 | 26 | 84 | 1 | 0 | 0 | 0 |
| E_40 | 75.68 | 0.9 | 27 | 83 | 1 | 0 | 0 | 0 |
| E_14 | 69.37 | 55.86 | 34 | 15 | 61 | 0 | 1 | 0 |
| E_2 | 65.77 | 1.8 | 38 | 71 | 2 | 0 | 0 | 0 |
| E_43 | 64.86 | 0 | 39 | 72 | 0 | 0 | 0 | 0 |
| E_42 | 63.96 | 0 | 40 | 71 | 0 | 0 | 0 | 0 |
| E_27 | 61.26 | 3.6 | 43 | 64 | 4 | 0 | 0 | 0 |
| E_7 | 56.76 | 0.9 | 48 | 62 | 1 | 0 | 0 | 0 |
| E_16 | 56.76 | 0 | 48 | 63 | 0 | 0 | 0 | 0 |
| E_4 | 54.95 | 2.7 | 50 | 58 | 3 | 0 | 0 | 0 |
| E_44 | 49.55 | 0.9 | 56 | 54 | 1 | 0 | 0 | 0 |
| E_3 | 43.24 | 0.9 | 63 | 47 | 1 | 0 | 0 | 0 |
| E_6 | 40.54 | 0 | 66 | 45 | 0 | 0 | 0 | 0 |
| E_47 | 36.94 | 0 | 70 | 41 | 0 | 0 | 0 | 0 |
| E_15 | 29.73 | 3.6 | 78 | 29 | 4 | 0 | 0 | 0 |
| E_25 | 29.73 | 1.8 | 78 | 31 | 2 | 0 | 0 | 0 |
| E_31 | 26.13 | 0.9 | 82 | 28 | 0 | 1 | 0 | 0 |
| E_9 | 26.13 | 0.9 | 82 | 28 | 1 | 0 | 0 | 0 |
| E_13 | 24.32 | 1.8 | 84 | 25 | 2 | 0 | 0 | 0 |
| E_21 | 24.32 | 0.9 | 84 | 26 | 1 | 0 | 0 | 0 |
| E_19 | 22.52 | 0 | 86 | 25 | 0 | 0 | 0 | 0 |
| E_20 | 21.62 | 0.9 | 87 | 23 | 1 | 0 | 0 | 0 |
| E_23 | 18.92 | 0 | 90 | 21 | 0 | 0 | 0 | 0 |
| E_34 | 0 | 0 | 111 | 0 | 0 | 0 | 0 | 0 |
| E_41 | 0 | 0 | 111 | 0 | 0 | 0 | 0 | 0 |
| E_18 | 0 | 0 | 111 | 0 | 0 | 0 | 0 | 0 |
| E_26 | 0 | 0 | 111 | 0 | 0 | 0 | 0 | 0 |

**Supplementary Table 2**. Genome completeness and contamination estimates for the GroopM binning of the synthetic dataset. The completeness estimate is based on the proportion of 111 universal single copy maker genes found in the bin. The contamination estimate indicates the multiplicity of these genes.

| **Unique ID** | **Summary statistics** | | **Single copy gene counts** | | | | | |
| --- | --- | --- | --- | --- | --- | --- | --- | --- |
|  | **Completeness** | **Contamination** | **0** | **1** | **2** | **3** | **4** | **5+** |
| GM_5 | 95.5 | 0.9 | 5 | 105 | 1 | 0 | 0 | 0 |
| GM_17 | 95.5 | 1.8 | 5 | 104 | 2 | 0 | 0 | 0 |
| GM_10 | 94.59 | 1.8 | 6 | 103 | 2 | 0 | 0 | 0 |
| GM_35 | 94.59 | 1.8 | 6 | 103 | 2 | 0 | 0 | 0 |
| GM_12 | 93.69 | 2.7 | 7 | 101 | 3 | 0 | 0 | 0 |
| GM_31 | 93.69 | 0.9 | 7 | 103 | 1 | 0 | 0 | 0 |
| GM_4 | 93.69 | 1.8 | 7 | 102 | 2 | 0 | 0 | 0 |
| GM_26 | 93.69 | 0.9 | 7 | 103 | 1 | 0 | 0 | 0 |
| GM_40 | 92.79 | 1.8 | 8 | 101 | 2 | 0 | 0 | 0 |
| GM_7 | 92.79 | 0.9 | 8 | 102 | 1 | 0 | 0 | 0 |
| GM_146 | 92.79 | 1.8 | 8 | 101 | 2 | 0 | 0 | 0 |
| GM_3 | 92.79 | 7.21 | 8 | 95 | 6 | 2 | 0 | 0 |
| GM_109 | 91.89 | 4.5 | 9 | 97 | 5 | 0 | 0 | 0 |
| GM_78 | 91.89 | 1.8 | 9 | 100 | 2 | 0 | 0 | 0 |
| GM_18 | 91.89 | 1.8 | 9 | 100 | 2 | 0 | 0 | 0 |
| GM_28 | 91.89 | 0.9 | 9 | 101 | 1 | 0 | 0 | 0 |
| GM_54 | 90.09 | 0.9 | 11 | 99 | 1 | 0 | 0 | 0 |
| GM_27 | 90.09 | 4.5 | 11 | 95 | 5 | 0 | 0 | 0 |
| GM_1 | 88.29 | 0.9 | 13 | 97 | 1 | 0 | 0 | 0 |
| GM_32 | 86.49 | 3.6 | 15 | 92 | 4 | 0 | 0 | 0 |
| GM_68 | 86.49 | 1.8 | 15 | 94 | 2 | 0 | 0 | 0 |
| GM_14 | 83.78 | 3.6 | 18 | 89 | 4 | 0 | 0 | 0 |
| GM_29 | 81.98 | 0.9 | 20 | 90 | 1 | 0 | 0 | 0 |
| GM_69 | 80.18 | 0.9 | 22 | 88 | 1 | 0 | 0 | 0 |
| GM_73 | 79.28 | 0.9 | 23 | 87 | 1 | 0 | 0 | 0 |
| GM_23 | 79.28 | 5.41 | 23 | 82 | 6 | 0 | 0 | 0 |
| GM_59 | 76.58 | 0.9 | 26 | 84 | 1 | 0 | 0 | 0 |
| GM_41 | 75.68 | 0.9 | 27 | 83 | 1 | 0 | 0 | 0 |
| GM_8 | 75.68 | 0.9 | 27 | 83 | 1 | 0 | 0 | 0 |
| GM_19 | 66.67 | 0.9 | 37 | 73 | 1 | 0 | 0 | 0 |
| GM_13 | 65.77 | 1.8 | 38 | 71 | 2 | 0 | 0 | 0 |
| GM_112 | 63.06 | 0.9 | 41 | 69 | 1 | 0 | 0 | 0 |
| GM_55 | 61.26 | 3.6 | 43 | 64 | 4 | 0 | 0 | 0 |
| GM_9 | 58.56 | 0.9 | 46 | 64 | 1 | 0 | 0 | 0 |
| GM_120 | 54.95 | 2.7 | 50 | 58 | 3 | 0 | 0 | 0 |
| GM_51 | 30.63 | 0.9 | 77 | 33 | 1 | 0 | 0 | 0 |
| GM_11 | 29.73 | 1.8 | 78 | 31 | 2 | 0 | 0 | 0 |
| GM_58 | 29.73 | 3.6 | 78 | 29 | 4 | 0 | 0 | 0 |
| GM_37 | 26.13 | 0.9 | 82 | 28 | 1 | 0 | 0 | 0 |
| GM_47 | 26.13 | 0.9 | 82 | 28 | 0 | 1 | 0 | 0 |
| GM_38 | 25.23 | 0 | 83 | 28 | 0 | 0 | 0 | 0 |
| GM_82 | 24.32 | 1.8 | 84 | 25 | 2 | 0 | 0 | 0 |
| GM_36 | 21.62 | 0.9 | 87 | 23 | 1 | 0 | 0 | 0 |
| GM_36 | 21.62 | 0.9 | 87 | 23 | 1 | 0 | 0 | 0 |
| GM_20 | 21.62 | 0.9 | 87 | 23 | 1 | 0 | 0 | 0 |
| GM_33 | 19.82 | 0 | 89 | 22 | 0 | 0 | 0 | 0 |
| GM_74 | 14.41 | 0.9 | 95 | 15 | 1 | 0 | 0 | 0 |
| GM_48.c | 11.71 | 0.9 | 98 | 12 | 1 | 0 | 0 | 0 |
| GM_65 | 5.41 | 0 | 105 | 6 | 0 | 0 | 0 | 0 |
| GM_91 | 0.9 | 0 | 110 | 1 | 0 | 0 | 0 | 0 |
| GM_93 | 0 | 0 | 111 | 0 | 0 | 0 | 0 | 0 |
| GM_150 | 0 | 0 | 111 | 0 | 0 | 0 | 0 | 0 |
| GM_75 | 0 | 0 | 111 | 0 | 0 | 0 | 0 | 0 |
| GM_42 | 0 | 0 | 111 | 0 | 0 | 0 | 0 | 0 |

**Supplementary Table 3**. A comparison of results for the GroopM and TF-ESOM binning methods using the synthetic dataset. The distribution of contig assignments shows the total number of contigs binned correctly, incorrectly or not assigned to any bin. The corresponding number of assembled bases for each cell is given in parentheses. GroopM incorrectly-binned far fewer long contigs (>5 Kbp) then TF-ESOM, while also correctly binning over 1900 more contigs than TF-ESOM. Note that the combined lengths of the extra correctly-binned contigs is ~18Mbp indicating that the majority of the extra contigs binned by GroopM are relatively short.

|  | **Total**  **number of Bins** | **Distribution of contig assignments** | | | **Contigs incorrectly assigned grouped by length (Kbp)** | | | | | |
| --- | --- | --- | --- | --- | --- | --- | --- | --- | --- | --- |
|  |  | **Correctly assigned** | **Incorrectly assigned** | **Unassigned** | **< 2** | **2 – 5** | **5 – 10** | **10 – 100** | **> 100** | **Longest**  **incorrect** |
| **GroopM** | 53 | 4,511  (130,450,045 bp) | 266  (597,197 bp) | 891  (2,684,491 bp) | 204 | 41 | 12 | 9 | 0 | 29,155 bp |
| **TF-ESOM** | 46 | 2,605 (112,655,005 bp) | 321  (11,571,818 bp) | 2,742  (9,504,910 bp) | N/A | 128 | 62 | 100 | 31 | 866,245 bp |

**Supplementary Table 4**. Genome completeness and contamination estimates for the Sharon et al. binning of the human gut microbiome dataset. The completeness estimate is based on the proportion of 111 universal single copy maker genes found in the bin. The contamination estimate indicates the multiplicity of these genes. The descriptions match those given at the source website: http://ggkbase.berkeley.edu/carrol/organisms

| **Unique ID** | **Description** | **Summary statistics** | | **Single copy gene counts** | | | | | |
| --- | --- | --- | --- | --- | --- | --- | --- | --- | --- |
|  |  | **Completeness** | **Contamination** | **0** | **1** | **2** | **3** | **4** | **5+** |
| CAREFA | *Enterococcus faecalis* | 94.59 | 4.5 | 6 | 100 | 4 | 1 | 0 | 0 |
| CARPRO | *Propionibacterium* sp. | 88.29 | 1.8 | 13 | 96 | 2 | 0 | 0 | 0 |
| CARSAU | *Staphylococcus aureus* | 83.78 | 2.7 | 18 | 90 | 3 | 0 | 0 | 0 |
| CARSEP3 | *Staphylococcus epidermidis* strain 3 | 82.88 | 2.7 | 19 | 89 | 3 | 0 | 0 | 0 |
| CARSHO | *Staphylococcus hominis* | 82.88 | 3.6 | 19 | 88 | 4 | 0 | 0 | 0 |
| CARSEP | *Staphylococcus epidermidis* strain 1 | 77.48 | 2.7 | 25 | 83 | 3 | 0 | 0 | 0 |
| CARPEP | *Peptoniphilus* sp. Carrol | 75.68 | 0.9 | 27 | 83 | 1 | 0 | 0 | 0 |
| CARSLU | *Staphylococcus lugdunensis* | 66.67 | 2.7 | 37 | 71 | 3 | 0 | 0 | 0 |
| CARLCI | *Leuconostoc citreum* | 53.15 | 0.9 | 52 | 58 | 1 | 0 | 0 | 0 |
| CARCAL | *Candida albicans* | 46.85 | 11.71 | 59 | 39 | 11 | 2 | 0 | 0 |
| CARFMA | *Finegoldia magna* | 38.74 | 0.9 | 68 | 42 | 1 | 0 | 0 | 0 |
| CARSTR | *Streptococcus* sp. | 14.41 | 0.9 | 95 | 15 | 1 | 0 | 0 | 0 |
| CARANA | *Anaerococcus* sp. | 4.5 | 0.9 | 106 | 4 | 1 | 0 | 0 | 0 |
| CARPAC | *Propionibacterium* acnes | 2.7 | 0 | 108 | 3 | 0 | 0 | 0 | 0 |
| CARSEP1P | *Staphylococcus epidermidis* strain 1 plasmids | 0.9 | 0 | 110 | 1 | 0 | 0 | 0 | 0 |
| ACDRDN | Redundant | 0 | 0 | 111 | 0 | 0 | 0 | 0 | 0 |
| CAREFAP | *Enterococcus faecalis* plasmid | 0 | 0 | 111 | 0 | 0 | 0 | 0 | 0 |
| CAREFAP2 | *Enterococcus faecalis* plasmid 2 | 0 | 0 | 111 | 0 | 0 | 0 | 0 | 0 |
| CARPROV | *Propionibacterium* sp. virus | 0 | 0 | 111 | 0 | 0 | 0 | 0 | 0 |
| CARSAU2 | *Staphylococcus aureus* strain 2 | 0 | 0 | 111 | 0 | 0 | 0 | 0 | 0 |
| CARSAUP | *Staphylococcus aureus* plasmid | 0 | 0 | 111 | 0 | 0 | 0 | 0 | 0 |
| CARSAUV | *Staphylococcus aureus* virus | 0 | 0 | 111 | 0 | 0 | 0 | 0 | 0 |
| CARSE4PL | *Staphylococcus epidermidis* strain 4 plasmids | 0 | 0 | 111 | 0 | 0 | 0 | 0 | 0 |
| CARSEMI | *Staphylococcus epidermidis* misc | 0 | 0 | 111 | 0 | 0 | 0 | 0 | 0 |
| CARSEP2 | *Staphylococcus epidermidis* misc. | 0 | 0 | 111 | 0 | 0 | 0 | 0 | 0 |
| CARSEP3P | *Staphylococcus epidermidis* strain 3 plasmids | 0 | 0 | 111 | 0 | 0 | 0 | 0 | 0 |
| CARSEP4 | *Staphylococcus epidermidis* strain 4 | 0 | 0 | 111 | 0 | 0 | 0 | 0 | 0 |
| CARSEPP | *Staphylococcus epidermidis* plasmid | 0 | 0 | 111 | 0 | 0 | 0 | 0 | 0 |
| CARSEPV | *Staphylococcus epidermidis* viruses | 0 | 0 | 111 | 0 | 0 | 0 | 0 | 0 |
| CARSEV13 | *Staphylococcus epidermidis* virus 013 | 0 | 0 | 111 | 0 | 0 | 0 | 0 | 0 |
| CARSEV14 | *Staphylococcus epidermidis* virus 014 | 0 | 0 | 111 | 0 | 0 | 0 | 0 | 0 |
| CARSEV46 | *Staphylococcus epidermidis* virus 4-6 | 0 | 0 | 111 | 0 | 0 | 0 | 0 | 0 |
| CARUNCL | Carrol unclustered | 0 | 0 | 111 | 0 | 0 | 0 | 0 | 0 |

**Supplementary Table 5**. Genome completeness and contamination estimates for the GroopM binning of the human gut microbiome dataset. The completeness estimate is based on the proportion of 111 universal single copy maker genes found in the bin. The contamination estimate indicates the multiplicity of these genes. The closest matching bins from the Sharon et al. assembly are indicated for each GroopM bin.

| **Unique ID** | **Corresponding Sharon et al. assembly ID(s)** | **Summary statistics** | | **Single copy gene counts** | | | | | |
| --- | --- | --- | --- | --- | --- | --- | --- | --- | --- |
|  |  | **Completeness** | **Contamination** | **0** | **1** | **2** | **3** | **4** | **5+** |
| GM_7 | CAREFA | 94.59 | 10.81 | 6 | 93 | 3 | 8 | 0 | 1 |
| GM_11 | CARPRO | 89.19 | 33.33 | 12 | 62 | 36 | 0 | 1 | 0 |
| GM_27 | CARPEP | 82.88 | 0.9 | 19 | 91 | 1 | 0 | 0 | 0 |
| GM_4 | CARSAU + CARSAUP | 76.58 | 9.91 | 26 | 74 | 7 | 4 | 0 | 0 |
| GM_76 | CARSEP + CARSEP3 | 75.68 | 4.5 | 27 | 79 | 4 | 1 | 0 | 0 |
| GM_75 | CARSHO | 72.97 | 3.6 | 30 | 77 | 4 | 0 | 0 | 0 |
| GM_17 | CARLCI | 63.96 | 1.8 | 40 | 69 | 2 | 0 | 0 | 0 |
| GM_3 | CARCAL | 54.05 | 23.42 | 51 | 34 | 22 | 4 | 0 | 0 |
| GM_6 | CARSTR + CARFMA | 49.55 | 12.61 | 56 | 41 | 13 | 0 | 1 | 0 |
| GM_9 | CARSLU | 36.04 | 0.9 | 71 | 39 | 1 | 0 | 0 | 0 |
| GM_66 | CARSEP1P + CARSEP3P | 18.02 | 0.9 | 91 | 19 | 1 | 0 | 0 | 0 |
| GM_19 | CARPAC | 13.51 | 0 | 96 | 15 | 0 | 0 | 0 | 0 |
| GM_31 | CARANA | 9.91 | 0.9 | 100 | 10 | 1 | 0 | 0 | 0 |
| GM_32 | CARSLU | 8.11 | 0.9 | 102 | 8 | 1 | 0 | 0 | 0 |
| GM_56 | CARSEP2 + CARSEP3P | 2.7 | 0 | 108 | 3 | 0 | 0 | 0 | 0 |
| GM_29 | Various CARSEP + CARSEP plasmids | 1.8 | 0 | 109 | 2 | 0 | 0 | 0 | 0 |
| GM_59 | Various CARSEP + CARSEP plasmids | 1.8 | 0 | 109 | 2 | 0 | 0 | 0 | 0 |
| GM_55 | CARCAL | 0.9 | 0 | 110 | 1 | 0 | 0 | 0 | 0 |
| GM_42 | CARSEV + CARSEV13 + CARSEV14 + CARSEV46 | 0.9 | 0 | 110 | 1 | 0 | 0 | 0 | 0 |
| GM_67 | CARSEP2 +CARSEV13 | 0 | 0 | 111 | 0 | 0 | 0 | 0 | 0 |
| GM_68 | CARSEP1P + CARSEPP | 0 | 0 | 111 | 0 | 0 | 0 | 0 | 0 |
| GM_33 | CARSAU + CARSAU2 + CARSAUV | 0 | 0 | 111 | 0 | 0 | 0 | 0 | 0 |
| GM_34 | NA | 0 | 0 | 111 | 0 | 0 | 0 | 0 | 0 |
| GM_38 | CAREFAP2 | 0 | 0 | 111 | 0 | 0 | 0 | 0 | 0 |
| GM_47 | Various CARSEP + CARSEP plasmids | 0 | 0 | 111 | 0 | 0 | 0 | 0 | 0 |

**Supplementary Table 6**. GroopM running time and memory usage. Benchmarking was performed on a DELL PowerEdge R815 server fitted with AMD Opteron 6174 (2200 MHz) CPUs. The Machine used in this benchmarking has 256Gb RAM.

| **Dataset** | **Performance statistics** | **GroopM stage** | | |
| --- | --- | --- | --- | --- |
|  |  | **Parse** | **Core** | **Recruit** |
| Synthetic data | Peak memory (MB) | 18150.6 | 494.9 | 52.2 |
|  | Average memory (MB) | 11945.5 | 281.2 | 52.2 |
|  | Time (mins) | 86.9 | 20.2 | 5.1 |
| Sharon et al. data | Peak memory (MB) | 5827.2 | 458.2 | 18.5 |
|  | Average memory (MB) | 4088.2 | 247.1 | 18.5 |
|  | Time (mins) | 92.4 | 10.2 | 5.1 |

**References**

Albertsen M, Hugenholtz P, Skarshewski A, Nielsen KL, Tyson GW, and Nielsen PH. 2013. Genome sequences of rare, uncultured bacteria obtained by differential coverage binning of multiple metagenomes. *Nature biotechnology* 31:533-538.

Bankevich A, Nurk S, Antipov D, Gurevich AA, Dvorkin M, Kulikov AS, Lesin VM, Nikolenko SI, Pham S, and Prjibelski AD. 2012. SPAdes: A new genome assembly algorithm and its applications to single-cell sequencing. *Journal of Computational Biology* 19:455-477.

Grubbs FE. 1950. Sample criteria for testing outlying observations. *The Annals of Mathematical Statistics* 21:27-58.

Hough PVC. 1962. Method and means for recognizing complex patterns. Google Patents.

Karlsson FH, Tremaroli V, Nookaew I, Bergström G, Behre CJ, Fagerberg B, Nielsen J, and Bäckhed F. 2013. Gut metagenome in European women with normal, impaired and diabetic glucose control. *Nature* 498:99-103.

Kohonen T. 1982. Self-organized formation of topologically correct feature maps. *Biological Cybernetics* 43:59-69.

Li H, and Durbin R. 2009. Fast and accurate short read alignment with Burrows–Wheeler transform. *Bioinformatics* 25:1754-1760.

Li H, Handsaker B, Wysoker A, Fennell T, Ruan J, Homer N, Marth G, Abecasis G, and Durbin R. 2009. The sequence alignment/map format and SAMtools. *Bioinformatics* 25:2078-2079.

Rousseeuw PJ. 1987. Silhouettes: a graphical aid to the interpretation and validation of cluster analysis. *Journal of computational and applied mathematics* 20:53-65.

Simpson JT, and Durbin R. 2010. Efficient construction of an assembly string graph using the FM-index. *Bioinformatics* 26:i367-i373.

Teeling H, Meyerdierks A, Bauer M, Amann R, and Glöckner FO. 2004. Application of tetranucleotide frequencies for the assignment of genomic fragments. *Environmental microbiology* 6:938-947.

Titus Brown C, Howe A, Zhang Q, Pyrkosz AB, and Brom TH. 2012. A Reference-Free Algorithm for Computational Normalization of Shotgun Sequencing Data.
